# Oral Treatment of Obesity by GLP-1 and Its Analogs

**DOI:** 10.3390/pharmaceutics17121596

**Published:** 2025-12-10

**Authors:** Natasa Holler, Ivana Ruseska, Anna-Laurence Schachner-Nedherer, Andreas Zimmer, Christina Petschacher

**Affiliations:** Institute of Pharmaceutical Sciences, Pharmaceutical Technology & Biopharmacy, University of Graz, 8010 Graz, Austria; natasa.holler@uni-graz.at (N.H.); ivana.ruseska@uni-graz.at (I.R.);

**Keywords:** glucagon-like peptide 1, oral peptide delivery, nanocarriers, nanoparticles, peptide therapeutics, gene delivery, targeted delivery, microbiome therapeutics, microbiome engineering, GLP-1 abuse

## Abstract

Obesity is a multifaceted disease that significantly increases the risk of various chronic conditions. GLP-1R (co)-agonists first emerged as therapeutics for treatment of type 2 diabetes mellitus and have since become an established drug class for improving glycemic control. The interest in GLP-1 for obesity treatment has surged in 2015 after the approval of Saxenda^®^ (liraglutide). To date, GLP-1 analogs are primarily administered by s.c. injection, which poses a significant burden on patient compliance. To address this challenge, research has focused on oral delivery. This review provides a concise overview of the techniques explored to enhance the oral delivery of GLP-1 analogs for the treatment of obesity. Relevant strategies include the following: (1) the use of permeation enhancers to increase gastrointestinal absorption of peptides; (2) micro- and nanocarriers loaded with GLP-1, including targeted delivery systems and general techniques for active drug targeting; (3) GLP-1 gene delivery; and (4) advanced microbiome systems for GLP-1 delivery. The potential for misuse and side-effects of GLP-1 analogs are also discussed.

## 1. Introduction

Obesity is a global health crisis characterized by excessive accumulation of body fat, which poses significant risks for a wide range of non-communicable diseases, including type 2 diabetes, cardiovascular disease, and certain cancers. According to the World Health Organization, more than a billion people worldwide are currently living with obesity with millions of them being children [1,2,3]. The postpandemic trends are especially concerning, showing an increasing prevalence in both childhood and adult overweight and obesity, with the expectancy of them being doubled in the next decade.

The traditional and simplest way of defining obesity is excess energy storage compared to energy used [4,5]. Nevertheless, the etiology of obesity is much more complex than this equation, given that the increasing prevalence can be related to a combination of multiple factors, such as genetics and epigenetics, environmental, cultural, and behavioral factors. It is essential that all these factors are studied in detail in order to prevent or treat obesity and obesity-related diseases effectively. Most commonly, obesity is defined as a body mass index [BMI (kg/m^2^)—dividing a person’s weight by the square of their height] greater than or equal to 30 [6]. Another measure used is the waist circumference, given that an increase in waist circumference can be related with increased mortality, even at a normal BMI [5]. Used together, BMI and waist circumference can provide better insight for distinguishing between elevated BMI due to high fat or high muscle content.

### 1.1. Physiology of Adipose Tissue and Pathophysiology of Obesity

Nowadays, it is known that multiple loci in the human genome are associated with adipogenesis and the physiological function of the adipose tissue. It is well established that the peroxisome proliferator-activated receptor gamma (PPAR-γ) is the master regulator of adipogenesis and the differentiation of pre-adipocytes to adipocytes. Worth noting is that there are several other factors (such as the C/EBPs, KLFs, PRDM16, and PGC-1α) steering the development of the adipose tissue. The expression levels of each factor determine the phenotypical characteristics of the adipose tissue [7].

Morphologically, the mammalian adipose tissue can be divided into two major types: white adipose tissue (WAT) and brown adipose tissue (BAT) [8]. White adipocytes are characterized as large spherical cells that store energy-providing lipids in the form of lipid droplets and are the main components of the WAT. This type of tissue is distributed in two anatomical compartments of the body: the subcutaneous and the visceral [9]. Obesity is characterized with ectopically expressed WAT, in areas such as mesenteric, omental and retroperitoneal fat. BAT, on the other hand, is more metabolically active than WAT due to the high mitochondrial activity, and functions by dissipating energy through heat [10]. It is known that the activity of BAT slows down with aging. Nevertheless, there is a third type of adipocytes, the so-called beige (or brite) adipocytes. These are formed from white adipocytes upon cold exposure or induced by activation of the PPARγ receptor and they demonstrate a similar energy-dissipating activity as BAT. The balance of both types of adipose tissue is crucial for maintaining homeostasis.

The adipose tissue, however, is more complex than this—it is composed of more than just adipocytes, and it also contains endothelial cells, macrophages, neutrophils, lymphocytes, and fibroblasts. This makes the adipose tissue metabolically active, which is visible through the production of various biologically active substances (called adipokines) important for cellular signaling and maintenance of homeostasis: enzymes, lipid transport molecules, cytokines, and complement-related proteins. Some of the most important substances secreted by the adipose tissue are hormones, such as adiponectin, an insulin-sensitivity regulator [11].

Impaired adipogenesis and dysfunction of BAT are well-established contributors to obesity and its associated comorbidities, including insulin resistance, type 2 diabetes mellitus (T2DM), inflammation, and cardiovascular disease. In obesity, adipose cells lose their ability to store excess lipids effectively, leading to ectopic fat deposition in organs such as the liver and muscles. This ectopic fat accumulation impairs cellular function and exacerbates metabolic dysregulation. Subcutaneous adipose tissue in obesity often experiences reduced vascularization, resulting in hypoxia. This hypoxic environment promotes fibrogenesis and the recruitment of macrophages, further perpetuating tissue inflammation [7,12]. The chronic inflammatory state, driven by the secretion of tumor necrosis factor α (TNFα) and other pro-inflammatory cytokines, disrupts insulin signaling pathways, causing insulin resistance. Over time, these metabolic and inflammatory changes increase the risk of T2DM, atherosclerosis, and other obesity-related conditions [2,7].

### 1.2. Treatments of Obesity

The primary objective of obesity treatment is weight loss, and this parameter is also established as a first measure of therapy efficacy when comparing different treatments. However, it is important to note that weight loss alone does not necessarily result in health benefits. Currently, more importance is given to loss of excessive fat specifically, while preserving or increasing muscle mass in obese individuals [13]. The aforementioned parameters, BMI and waist circumference, are the main readily available parameters. However, a whole range of other metabolic parameters, such as plasma triglycerides, cholesterol, Hb1Ac, inflammation status etc. can also be taken into account when evaluating a particular obesity treatment and its benefits [13,14].

As outlined by WHO, the main strategies in the obesity management encompass healthy diet, physical activity, and medical and surgical interventions [15]. The initial two measures belong to conservative management of obesity, which entails dietary interventions (such as lowering the caloric intake) together with physical activity and behavioral therapy, and they necessitate the intervention of clinicians, psychologists, and dietitians [5]. The latter two interventions encompass (1) bariatric and metabolic procedures (bariatric surgery and endoscopic procedures) and (2) pharmacotherapy of obesity. These interventions will be discussed briefly in the following text.

#### 1.2.1. Bariatric and Metabolic Procedures

A total of approximately 480,000 bariatric surgeries were performed worldwide during the period from 2021 to 2022, of which approximately 60% were the sleeve gastrectomy (SG). The second most frequently performed intervention was Roux-Y gastric bypass (RYGB), accounting for approximately 30% of the total number. The median initial BMI of patients undergoing primary bariatric surgery was between 40 and 45 kg/m^2^ in most registries but depended strongly on the presence of metabolic comorbidities [16]. In terms of the mechanisms underlying weight reduction, the procedures, such as sleeve gastrectomy and adjustable gastric band are based on the calorie restriction due to the reduced volume of the stomach [17]. On the other hand, bypassing the duodenum together with the first portion of the jejunum as in RYGB; or duodenum, jejunum, and the first tract of ileum, as in biliopancreatic diversion, is associated with malabsorption of nutrients. However, only the latter effects are also associated with improvements in obesity-related metabolic complications [18]. It was shown that the bypassing of the stomach and first part of the small intestine increases the insulin sensitivity promptly and independently of the caloric restriction and weight loss, which is why those procedures are also called metabolic surgeries [17]. Similar classification can be found for the endoscopic bariatric and metabolic therapies (EBMTs), which have emerged nowadays as less invasive alternatives to bariatric surgeries [17,19]. The primary efficacy endpoint for gastric interventions (e.g., intragastric balloons and endoscopic gastric remodeling) is weight loss, whereas the primary efficacy endpoint for small-bowel interventions (e.g., duodenal-jejunal bypass liner and duodenal mucosal resurfacing) is glycemic improvement with weight loss as co-primary or secondary objective [19].

Speaking of efficacy, the RYGB tends to provide greater excessive weight loss after 5 years of follow up compared to the SG [20,21,22], but it is also associated with a higher complication rate comparing to SG [23,24]. The postprandial secretion of gut hormones, glucagon-like peptides (GLP-1, GLP-2), and PYY, is postoperatively more enhanced after RYGB than after SG, whereas the secretion of glucose-dependent insulinotropic polypeptide (GIP) is blunted after RYGB [25]. This strengthens the hypothesis that metabolic factors such as incretin disorders, and not solely gluttony, play a more significant role in the pathogenesis of obesity. The better understanding of these processes opens opportunities for more efficient and individualized obesity therapies, as was demonstrated in late success of therapies with GLP-1 agonists [25,26].

#### 1.2.2. Pharmacotherapy

Pharmacotherapy is recommended for individuals with BMI ≥ 30 or BMI ≥ 27 in combination with weight-related complications (high blood pressure, type 2 diabetes or abnormal levels of fat in the blood) and when the response to lifestyle-based treatment is unsatisfactory [6].

There are currently several drugs on the market for the treatment of obesity. Some of these, such as the amphetamine derivatives phentermine, amfepramone (diethylpropion), benzphetamine, phendimetrazine, and mazindol, are approved by FDA but only for short-term use (<12 weeks) [6,27]. However, according to the information found on drugs@FDA [28], most of the products containing these drugs are already discontinued from the market. Furthermore, due to the cardiovascular adverse effects and an unfavorable risk/benefit balance, EMA has withdrawn approvals for some of these drugs, such as for phentermine in 2000 and diethylpropion (amfepramone) in 2023 [29].

For long-term use (>12 weeks), two drug combinations have been approved in the USA and some EU-countries: phentermine with topiramate (GABA receptor agonist/glutamate receptor antagonist) (Qsymia^®^ in USA or Qsiva^®^ in Europe, Vivus, Inc., Campbell, CA, USA), and naltrexone with bupropion (opioid receptor antagonist/noradrenaline-dopamine uptake-reuptake inhibitor), (Contrave^®^ in USA, Mysimba^®^ in Europe, Nalpropion Pharmaceuticals LLC, Morristown, NJ, USA) [30,31,32,33]. However, in September 2023, EMA began revising the use of Mysimba^®^ due to concerns regarding the potential long-term cardiovascular risk. Although the current consensus is that the benefits of Mysimba outweigh its risks, the final results of ongoing safety studies are expected in 2028 [33,34,35].

Other antiobesity medicines on the market are orlistat (Xenical^®^, CHEPLAPHARM Arzneimittel GmbH, Greifswald, Germany; Alli^®^, Haleon Ireland Dungarvan Ltd, Dungarvan, Co. Waterford, Ireland), which prevents dietary fat absorption in intestinal lumen due to the irreversible inhibition of gastric and pancreatic lipases, diacylglycerol lipase (DAGL), and αβ-hydrolase 12 (ABHD12). It has also been approved by the FDA as an OTC drug in a lower dosage (60 mg, Alli^®^), while the higher dosage package (120 mg, Xenical^®^) is a prescription drug [28,36]. Preventing fat absorption and the satiating effect can be also based on a physical mechanism alone, as in the case of medical products containing various organic polymers that act as fiber supplements (e.g., Formoline^®^, Certmedica International GmbH, Aschaffenburg, Germany; and Plenity^®^, Gelesis, Inc., Boston, MA, USA). However, there is still a lack of evidence for a significant clinical effect on body weight [37].

Setmelanotide (Imcivree^®^, Rhythm Pharmaceuticals, Inc., Boston, MA, USA) is a melanocortin-4 receptor (MC4R) agonist indicated only for the treatment of obesity caused by deficiency in MC4R signaling. This deficiency causes the disruption of signaling in the hypothalamus, leading to overeating and extreme early onset of obesity. Setmelanotide was approved by the FDA in 2020 for patients aged 6 years and older and it also received orphan drug status in Europe in 2021. There are also clinical trials in progress for the potential use of setmelanotide in the treatment of acquired hypothalamic obesity, which can be seen as hypothalamic substitution therapy (e.g., following tumor treatment in hypothalamic region) [38,39,40,41]. Moreover, an oral formulation of MC4R agonist under the name LB54640 is currently in phase 2 clinical studies (NCT06046443).

The FDA and EMA have approved until now three GLP-1 receptor agonists for treatment of obesity: liraglutide (2015, Novo Nordisk), semaglutide (2021, Novo Nordisk), and tirzepatide (2022, Eli Lilly). Prior to these approvals, liraglutide and semaglutide were in use for treatment of diabetes mellitus type 2 (T2DM).

Liraglutide as monotherapy is currently available on the market as s.c. injection Victoza^®^ for the treatment of diabetes (maximal dosage 1.8 mg once daily) and as Saxenda^®^ for the treatment of obesity (maximal dosage 3.0 mg once daily, Novo Nordisk A/S, Bagsværd, Denmark. It also has an orphan designation for Wolfram disease, a rare genetic disorder characterized by juvenile-onset diabetes mellitus, diabetes insipidus, optic nerve atrophy, hearing loss, and neurodegeneration [42,43,44].

Semaglutide is marketed in the form of an s.c. injection as Ozempic^®^ for treatment of T2DM (maximal dosage 1 mg once weekly) and as Wegovy^®^ for treatment of obesity, metabolic associated steatohepatitis (MASH), and for reducing of cardiovascular (CV) risk in patients with a CV disease and obesity or are overweight (maximal dosage 2.4 mg applied once weekly, Novo Nordisk A/S, Bagsværd, Denmark [45,46]. It has also been approved by the FDA (2019) and EMA (2020) in a tablet form (Rybelsus^®^), but currently only for treatment of T2DM [28,47]. In December 2024, the company (Novo Nordisk) acquired approval by FDA for an additional formulation of Rybelsus^®^, which reduces the necessary amount of semaglutide [48]. In the original formulation (designated as R1), the listed excipients were magnesium stearate, microcrystalline cellulose, povidone, and salcaprozate sodium (SNAC, sodium N-[8-(2-hydroxybenzoyl)amino]caprylate), which is used as absorption enhancer. In the first approval of Rybelsus^®^, the available dosages were 3 mg, 7 mg, and 14 mg, whereas the maximal maintenance dosage was 14 mg of semaglutide (once daily). Meanwhile, the recently approved formulation (R2 formulation) contains, besides the active substance, only SNAC and magnesium stearate. It provides the corresponding bioequivalence with reduced amount of drug, which is 1.5 mg, 4 mg, and 9 mg of semaglutide, respectively [48]. The EMA approved the alternative new formulation already in August 2024, followed by an approval of two additional dosages in September 2024, namely 25 mg and 50 mg of semaglutide for a once-daily application. The semaglutide in these dosages is, however, still only approved for T2DM therapy and it is currently available only in the latter formulation [49,50].

Tirzepatide is the first approved dual agonist in the entero-pancreatic hormone regulation. It is an GLP-1 receptor and glucose-dependent insulinotropic peptide (GIP) dual agonist. It was approved by EMA as Mounjaro^®^ (s.c. injections, Novo Nordisk A/S, Bagsværd, Denmark) for treatment of both T2DM and obesity [51], whereas it is approved by the FDA as Mounjaro^®^ only for treatment of T2DM and separately as Zepbound^®^ for treatment of obesity [45,46]. The maximal approved maintenance dosage for both products is 15 mg per week. In December 2024, the therapeutic indication of both Mounjaro^®^ and Zepbound^®^ was extended to treatment of the moderate to severe obstructive sleep apnea (OSA) in adults with obesity [52,53].

### 1.3. Physiology of GLP-1

#### 1.3.1. Systemic Effects of GLP-1: From Physiology to Pharmacology

**Figure 1:**
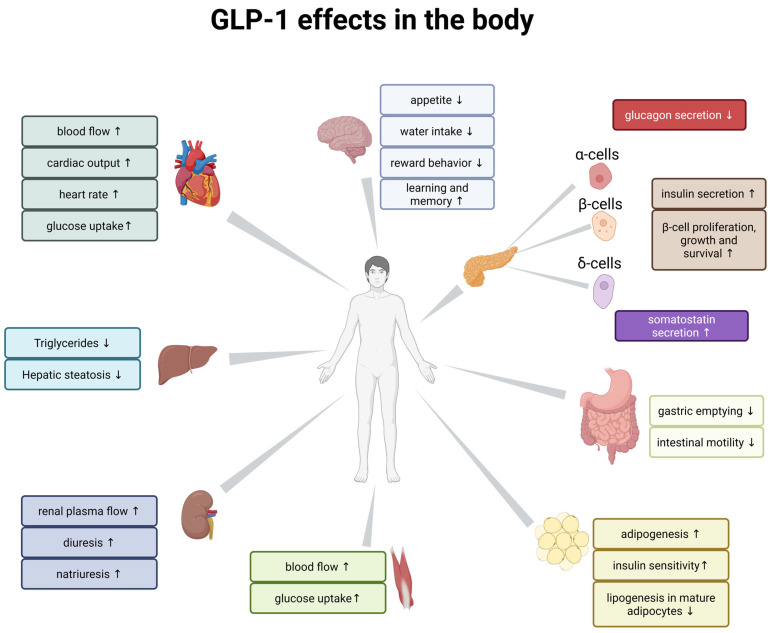
Systemic effects of GLP-1. Created in BioRender. Holler, N. (2025). https://BioRender.com/5ozl7s0.

GLP-1 is a 30-amino acid peptide hormone released from intestinal epithelial L-cells in response to meal uptake [54]. Due to its contributions to limit postprandial glucose excursions (incretin effect), it emerged as a target for type-2 diabetes treatment [54,55]. However, recent years have witnessed a mounting body of research on the effects of GLP-1 analogs, demonstrating the systemic effects of GLP-1 which extend beyond the incretin effect and affect diverse organ systems (see Figure 1). A comprehensive summary of the effects of GLP-1 on the different organs or functions within the body can be found elsewhere [56].

The effect of GLP-1 on enhancing insulin secretion from pancreatic β-cells only at a certain glucose threshold is well understood and widely accepted [57,58,59]. Furthermore, GLP-1 has been found to have positive effects on β-cell proliferation, growth, and survival. It improves glucose sensitivity in glucose-resistant β-cells [58,60,61,62]. GLP-1 inhibits glucagon secretion from pancreatic α-cells, a process that has been demonstrated to be deficient in diabetic patients in both, hypo- and hyperglycemic conditions [63]. However, the nature of its effect on glucagon secretion (whether it is a direct effect on α-cells or an indirect effect via stimulation of insulin and somatostatin secretion from β- and δ-cells, respectively) is still being debated [54,57,64,65]. GLP-1 stimulates gastric accommodation, a process of relaxing the stomach in anticipation of food intake; delays gastric emptying; and decreases intestinal motility [66,67]. GLP-1R is expressed in many areas of the brain. This may explain the diverse central effects of GLP-1 on appetite regulation (suppression of food intake), body weight and temperature, malaise, fluid homeostasis, stress response, and learning and memory. GLP-1 also modulates hedonic or non-homeostatic feeding by acting on neural circuits involved in reward behavior, motivation, and addiction [56,68].

As early research focused primarily on the physiological actions of native GLP-1, the approval of GLP-1 analogs led to the recognition of additional effects that can be attributed to GLP-1R activation. In the liver, the long-term effects of GLP-1 analogs generally include a decrease in the content of triglycerides in hepatocytes, and an amelioration of hepatic steatosis. In the kidney, the GLP-1R agonists stimulate diuresis and natriuresis comparable to conventional diuretics leading to a decrease in arterial blood pressure [69].

According to both in vivo and in vitro studies on related cell-lines, GLP-1 has direct effects on adipose and muscle tissue too. In the adipose tissue, GLP-1 analog exendin-4 has been shown to increase the secretion of anti-inflammatory adiponectin from the visceral omental fat [70]. In vitro studies on 3T3-L1 cells demonstrated that the GLP-1 analog liraglutide promotes adipogenesis of pre-adipocytes, whereas it inhibits lipogenesis in mature adipocytes [71,72]. Consequently, the adipocyte hyperplasia resulting from pre-adipocyte adipogenesis has been shown to increase the insulin sensitivity [72]. In muscle tissue, GLP-1 has been observed to enhance glucose uptake under normoglycemic conditions [73]. GLP-1 also promotes vasodilation in adipose and muscle tissue, and consequently, it increases microvascular blood flow and blood volume in skeletal and cardiac muscles. Finally, GLP-1 has been demonstrated to increase cardiac output and heart rate. It is evident that the aforementioned factors result in enhanced tissue delivery of oxygen, nutrients and insulin. This, in turn, leads to improved metabolic function of muscle tissue and its response to glucose [74,75].

#### 1.3.2. Regulation of GLP-1 Expression

**Figure 2:**
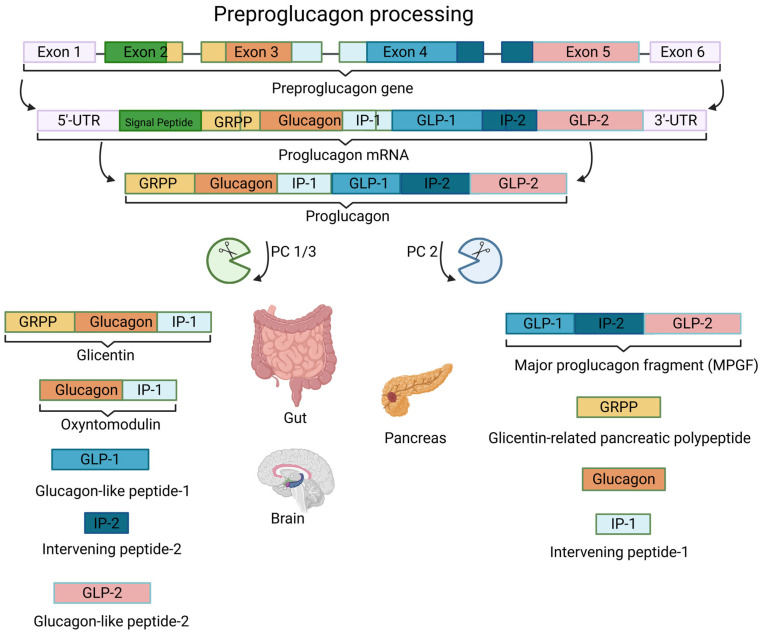
Processing of the preproglucagon gene to GLP-1. Created in BioRender.com. Holler, N. (2025). https://BioRender.com/zmznx0i. Layout adapted from Lafferty et al., 2021 [78], licensed under CC BY 4.0.

The GLP-1 gene is a part of the preproglucagon-gene (*Gcg*), which is expressed in a specific population of enteroendocrine cells (L-cells), pancreatic α-cells, and in certain neurons in the NTS (nucleus of the solitary tract) in the brain stem [54,66]. The expression of *Gcg* is regulated by many factors, ranging from nutrients to hormones. Moreover, the effect of a single signal molecule on gene expression can vary depending on the cell type it acts on. For instance, insulin increases the *Gcg* expression in the pancreatic α-cells, whereas in the enteroendocrine L-cells, it provokes an opposite effect. After gene expression, the post-translational processing involves the specific cutting of 160 amino acids long preproglucagon, firstly to proglucagon by enzymes signal peptidase, and thereafter, to different proteohormones by tissue-specific enzymes called prohormone convertase (PC) (see Figure 2) [54,56,76]. In this manner, a fine-tuning of the information encoded in *Gcg* is enabled, depending on the cell type-specific, co-expressed PC subtypes. For example, in the enteroendocrine L-cells and some specific neurons in NTS, where PC 1/3 activity is predominant, the result of post-translational modification is the GLP-1, GLP-2, oxyntomodulin, glicentin and IP2. In an opposite manner, in the pancreatic α-cells, due to the high PC2 activity, among other peptides, the glucagon will be produced [77]. This sheds light on how complex, systematic, and highly precise the regulation of the glucose homeostasis is.

There are several forms of GLP-1 that can be derived from proglucagon and they mainly differ in their insulinotropic capacity and their relative abundance in particular species. Three main forms include GLP-1(13–7) and two truncated forms, namely GLP-1(7–37) and GLP-1(7–36)amide, also termed as glycine-extended GLP-1 and amidated GLP-1, respectively [56]. In the human body, the predominant circulating GLP-1 form is GLP-1(7–36) amide, and it is generally referred to as GLP-1 when no further specific designation is provided [54,79,80].

#### 1.3.3. GLP-1 Receptor (GLP-1R)

GLP-1 exerts its effects via the GLP-1R, which is a member of class B1 of G-protein coupled receptors (GPCRs), also known as the secretin family, along with receptors for other peptide hormones such as glucagon, secretin, GIP, etc. [57,81]. Like all receptors of the GPRC family, the B1 subfamily receptors have a structure with seven transmembrane helices, where the transmembrane helices are connected outside the cell by three extracellular loops (ECL1-3) and inside the cell by three intracellular loops (INCL1-3). The B1 subfamily is also characterized by a “two-domain binding model” in which both the transmembrane domain (TMD) and the extracellular domain (ECD) are involved in ligand recognition [81,82,83]. First, the ECD rapidly binds to the C-terminus of the ligand. This represents the initial receptor-ligand specificity. Subsequently, the N-terminus of the ligand enters the allosteric pocket of the TMD, causing changes in the receptor conformation and allowing the recruitment of G-proteins on the intracellular side. This determines the rate of receptor activation [81,83].

#### 1.3.4. Degradation of GLP-1

The half-life of native GLP-1 in the body is approximately 1–2 min after the secretion owing primarily to the enzymatic degradation by dipeptidylpeptidase-4 (DPP-4) and the rapid renal elimination [56,84]. The DPP-4 is a ubiquitous exopeptidase that cleaves dipeptide sequence containing either proline or alanine in the penultimate position from the N-terminal end of the peptide. The DPP-4 cleavage of GLP-1 takes place between Ala8 and Glu9. In this manner, the DPP-4 generates the N-truncated GLP-1 forms, namely GLP-1(9–36)amide and GLP-1(9–37), which have no insulinotropic capacity. They still possess a certain affinity on the GLP-1 receptor, as the C-terminus is responsible for the binding to the ECD of the GLP-1R; however, they cannot activate the signaling cascade, because the N-terminal is crucial for the activation of second messengers and the signal transduction [85,86]. Therefore, they are considered as weak competitive antagonists [55,56,87].

Since the DPP-4 is present in the endothelial membranes of the gut capillaries, the degradation takes place already in the close proximity to the L-cells. Only 50% of the secreted GLP-1 can reach the general circulation. Further degradation happens in the liver and kidneys, where DPP-4 is also present as a membrane-bound ectoenzyme. Following a single pass through the liver bed, an additional 40% of the remaining GLP-1 is degraded. The DPP-4 degradation also occurs to a lesser extent in the blood, as DPP-4 is also present in a soluble form in the systemic circulation [84]. This raises the question of how much intact GLP-1 can reach the pancreas via the systemic circulation to induce an insulinotropic response [84]. Alternatively, recent studies on GLP-1R KO mice have revealed the importance of GLP-1 produced in the islets in regulating glucose levels after the *Gcg* gene was knocked down systemically and then selectively reactivated in either pancreatic or intestinal cells [88,89].

#### 1.3.5. Incretin Effect: Regulation of the Postprandial GLP-1 Secretion

In humans and other primates, the density of enteroendocrine L-cells increases along the intestine, with very few or no L-cells present in the duodenum, the highest concentration are found in the ileum and a considerable amount in the colon [54]. The L-cells are equipped with the microvilli and are in direct contact with the intestinal lumen, and, therefore, with nutrients. However, the extent to which nutrients can directly activate the initial GLP-1 secretion still remains unclear due to the existence of divergent opinions suggesting indirect neural, endocrine, or paracrine pathways of the GLP-1 response [77]. The majority of L-cells necessary for an early GLP-1 response are located in the distal intestines. In addition, studies with atropine (a specific M1 muscarinic-receptor antagonist) show that atropine diminishes and postpones the early GLP-1 response after OGT (oral glucose test). These findings substantiate the hypothesis that L-cells are under direct cholinergic regulation via the vagus nerve, rather than being stimulated by nutrients [66,90]. However, according to alternative opinions, even a small number of L-cells in the proximal jejunum may be sufficient for direct nutrient-stimulated GLP-1 release [54]. Therefore, it is more plausible that the combination of all these factors (nutrients, the autonomic nerve system, and the previous GIP secretion) acting in a synchronized manner is the necessary prerequisite for the rapid release of GLP-1 from the L-cells following ingestion [91].

Studies on humans reported a rapid GLP-1 release after the oral administration of glucose, which reaches its maximum after about 30 min and keeps this maximal level of around 300% of the basal GLP-1 level in a plateau phase for approximately an additional hour. A similar curve was observed for the GIP response, where the first peak was reached after 5 min and the second one during the plateau phase after around 45 min. The responses depended in both cases on the type of ingested nutrient [92].

GLP-1 and GIP are currently the only two known insulinotropic, gut-derived hormones, and are key mediators of the incretin effect [57,91,93]. While GIP increases the glucagon secretion, the GLP-1 suppresses it in hyperglycemic conditions, which leads to reduced gluconeogenesis in the liver. In a study that compared the insulinotropic effects of GLP-1(7–36 amide) with GIP after intravenous administration, it was shown that they contribute nearly equally to the incretin effect in healthy subjects [94]. It is believed that the function of those two incretin hormones might be attributed to their location. Since GIP is produced in K-cells mainly distributed in the proximal intestine (duodenum), its secretion is thought to be stimulated already with smaller amounts of more simple and faster absorbable transitioning nutrients [54]. The GIP release seems to be more dependent on the rate of nutrient absorption than on the nutrient presence per se, which can explain a reduced GIP response in individuals with intestinal malabsorption [91]. On the other hand, GLP-1 is mainly produced in the distal intestine and is associated with more complex nutrients and larger meals rich in fats and carbohydrates. The L-cells are also present more distally, in the colon, which suggests that the GLP-1 secretion correlates with meal size, as with larger meals more L-cells can be activated [54,91].

In patients with insulin resistance, it was observed that an impaired incretin response after a mixed meal is associated with both incretins, GLP-1 and GIP [95]. In diabetic patients, the insulinotropic effects of both incretins are diminished; however, the insulinotropic potential of GLP-1 is more preserved [96,97,98]. Consequently, the initial research on incretin mimetics focused only on GLP-1 and the strategies to enhance its efficacy for antidiabetic therapy [98,99]. Nevertheless, with the recent approval of tirzepatide for the treatment of obesity, the interest in GIP and other proglucagon-derived hormones has increased significantly [98].

## 2. Approved GLP-1 Analogs

**Figure 3:**
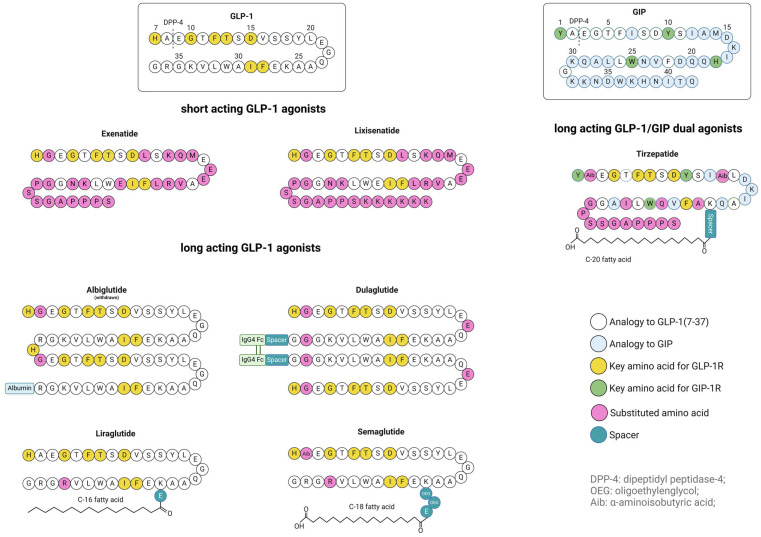
Molecular structures of the FDA-approved GLP-1R agonists and co-agonists. Created with BioRender.com using an adapted template. Holler, N. (2025). https://BioRender.com/5n7ehcf. Layout inspired by Yu et al., 2018 [55].

As was discussed in the previous chapter, the main issue with use of GLP-1 in the therapeutic purposes is its short half-life time due to the rapid degradation by DPP-4 and the rapid renal elimination. To overcome this, different modifications on the peptide structure were introduced to increase the receptor specificity and its signaling efficacy on the one side, and to increase the resistance against enzymatic degradation on the other side. However, only several GLP-1R agonists were approved and reached the market until now. Their peptide sequences including structure modifications are schematically presented in the Figure 3, whereas Table 1 summarizes the key physicochemical and clinical characteristics of approved GLP-1 analogs [55]. Of note, the dual GLP-1R/GIPR agonist tirzepatide is the only drug from the class of multiple incretin receptors co-agonists that has been approved to date. However, many such candidates are currently in the pipeline, and they will be the subject of the next chapter. With respect to the structural modifications, the focus will be exclusively on the approved GLP-1R (co-)agonists. The sole exception is albiglutide, which was initially approved in 2014 but subsequently withdrawn in 2017 [28,100,101]. The company has stated, however, that this decision was motivated by commercial factors, rather than concerns regarding the efficacy or safety of the medicine [102].

Exendin-4 is a GLP-1 analog found and isolated from the venom of the Gila monster (Heloderma suspectum) [103]. Exendin-4 shares only 53% homology with human GLP-1. However, it possesses a higher affinity for the ECD of GLP-1R in comparison to the native GLP-1. This can be explained by Exendin-4’s higher propensity for α-helix in solution, and stronger ionic interactions with the receptor in the peptide’s C-terminal region. Furthermore, the substitution of the Ala2 with glycine prevents the degradation by DPP-4. Exendin-4 is also extended with eight amino acid residues in C-terminal region [57]. It has been hypothesized that this additional sequence, designated as Trp-cage (due to its propensity to form in certain solvents a stable tertiary structure that envelops Trp25), may be responsible for the augmented affinity of exendin-4 for GLP-1R. Nonetheless, the radioligand competition studies by Runge et al. with chimeric and truncated peptides demonstrated that this plays a relatively minor role, thereby attributing greater significance to the divergent residues in the central part of exendin-4 [57,104].

The synthetic analog of exendin-4, exenatide, was the first FDA-approved GLP-1R agonist (2005 Byetta^®^, Astra Zeneca LP, Wilmington, DE, USA). Byetta^®^ is approved for s.c. administration in the dosages of 5 µg and 10 µg of exenatide twice daily as its terminal half-life is 2.4 h, and it is approved only for the treatment of T2DM [28,105]. Bydureon^®^ (AstraZeneca LP, Wilmington, DE, USA) was approved in 2012 and contains an extended-release formulation of exenatide for once-a-week administration at a dosage of 2 mg. The extended release was achieved by incorporation of the peptide into PLGA-microspheres consisting of 50:50 poly(D,L-lactide-co-glycolide) polymer. The powder mixture of microspheres and sucrose is either packed separately from the aqueous phase in a single-dose pen, and the patient must then resuspend the powder prior to injection (Bydureon^®^ pen); or it is packed as an oil suspension of microspheres in medium-chain triglycerides (MCTs), which facilitates the resuspension (Bydureon^®^ BCise™, AstraZeneca LP, Wilmington, DE, USA) [106,107]. Both, Byetta^®^ and Bydureon^®^, were discontinued from the US-market in October 2024; however, only due to the economic reasons, as the market exclusivity periods are expiring [108]. In November 2024, Amneal received FDA approval for the exenatide prefilled pen injection (Exenatide synthetic), which references Byetta^®^, representing the first generic drug of a GLP-1 analog [28,108,109].

Lixisenatide is an exendin-4 analog approved 2013 in Europe (Lyxumia^®^)), and 2016 in USA (Adlyxin^®^, both Sanofi-Aventis U.S. LLC, Bridgewater, NJ, USA) [28,110,111]. Same as exendin, it represents a short-acting GLP-1 receptor agonist that lowers postprandial glucose levels primarily by delaying gastric emptying [59]. It has a terminal mean half-life of approximately 3 h and is administered subcutaneously once daily before the first meal. The granted approvals are for 10 µg as the initial dose and 20 µg as the maintenance dose after 2 weeks [28,110]. Lixisenatide was developed using Structure Inducing Probe (SIP) technology, which aims to improve the stability of peptides by adding short peptide sequences at either the N- or C-terminus to achieve more ordered and stable conformations based on hydrogen bonding. Compared to the structure of exendin-4, the proline residue at position 36 was eliminated and six additional lysine residues were added to the C-terminus [112].

Dulaglutide, albiglutide, liraglutide, and semaglutide represent the long-acting GLP-1 analogs. They all possess some additional molecular structures that have been added to the peptide sequence to prolong the half-life of the resulting peptide conjugates. Although the nature of the modifications differed, the strategies employed to prolong the half-life of GLP-1 were similar and they all relied on the neonatal Fc receptor (FcRn). The FcRn receptor is the primarily responsible for the protecting of IgG from catabolic activity in the body. It maintains IgG homeostasis, which is essential for an appropriate immune response to foreign antigens. Similarly, FcRn can prolong the half-life of its second ligand, albumin [113,114]. Albumin is an attractive molecule to improve the pharmacokinetics and pharmacodynamics of the drug due to its inherent properties, namely its heart-shaped form, hydrophobic cavity, and due to its wide tissue distribution. The drug is usually either covalently coupled directly to albumin to form a drug-albumin construct (e.g., albiglutide) or designed to bind non-covalently to the endogenous albumin pool (e.g., via a long-chain fatty acid as in semaglutide and liraglutide, or via some smaller albumin-binding domains with a high affinity for albumin) [115,116,117]. In this manner, due to the intracellular FcRn recycling, the peptide is protected from hepatic metabolic degradation [114]. (For further details see Section 6.1.1).

Dulaglutide is an Fc fusion protein consisting of two DPP-4-protected GLP-1(7–37) sequences (also referred to as V8-GLP-1) linked at their C-termini to the N-terminus of the human Fc domain of immunoglobulin G4 (IgG4) by a glycine- and serine-based spacer [56,118,119]. The length and the structure of the spacer appeared to be critical for GLP-1 activity, whereas the Fc moiety was designed to bind the neonatal Fc receptor (FcRn), but to have a minimal (immunogenic) Fc activity [118,119]. Dulaglutide (Trulicity^®^, Eli Lilly and Company, Indianapolis, IN, USA) was approved in 2014 for once-weekly subcutaneous injection in 0.75 mg and 1.5 mg doses, and its half-life is around five days [28,120].

Liraglutide (Victoza^®^) was approved for the treatment of T2DM in 2010 and for the treatment of obesity in 2015 (Saxenda^®^, both Novo Nordisk A/S, Bagsværd, Denmark) [28,43,44]. The drug has a half-life of 13 h and is administered subcutaneously once daily. The initial dose is 0.6 mg, while the maintenance dose is 1.2 mg. Also, if additional glycemic control is required, the dose can be increased to 1.8 mg once daily, whereas for the treatment of obesity, it may reach up to 3 mg per day [28,43]. In liraglutide, to achieve a high receptor specificity while maintaining resistance to DPP-4, the GLP-1 sequence was modified at positions 26 and 34. In the position 26, a lysine residue was coupled via a γGlu linker to the palmitate (C16), while the lysine in position 34 was substituted by arginine for technical reasons to enable a semi-recombinant production of the liraglutide. In this way, the peptide backbone is produced by a recombinant process, whereas the attachment of the fatty acid takes place subsequently in a simple chemical reaction. The linker was introduced to compensate for the loss of the acidic group in palmitate used for amide linkage (the fatty acid is important for binding to albumin, for a detailed explanation see Section 6.1.1) [116,121]. Although the alanine at position 2 was retained, partial protection against rapid degradation by DPP-4 remained, either by reversible binding to albumin or by direct steric hindrance [116].

Semaglutide (Ozempic^®^, Wegovy^®^, Novo Nordisk A/S, Bagsværd, Denmark) has a half-life of about one week and is designed for once-weekly dosing [45,46]. After liraglutide, the idea was to develop a GLP-1 analog which would bind more loosely to albumin to increase the free peptide fraction, since only free peptide is available to the GLP-1R [116]. The key differences from liraglutide are the following: (1) the substitution of alanine at position 2 with aminoisobutyric acid (Aib) for resistance against DPP-4; (2) a γGlu-2xOEG (γ-Glutamyl-bis(oligoethylenglycol)) spacer for more peptide flexibility and an optimized binding to the receptor; and (3) stearic acid instead of palmitic acid, as stearic acid showed a higher binding affinity to albumin and the optimal overall properties following extensive characterization [116,122].

Albiglutide is a dimer of Gly8-modified GLP-1(7-37) coupled to albumin (HSA) at the C-terminus. The doubled GLP-1 sequence is chosen to increase the distance between albumin and the distal GLP-1. The glycine modification at position 8 increases resistance to DPP-4, but also decreases receptor affinity. The half-life achieved is approximately 5 days and the solution was approved for 2 doses, 30 mg, and 50 mg once weekly [28,100,101,116,123].

Tirzepatide (Mounjaro^®^, Zepbound^®^, Eli Lilly and Company, Indianapolis, IN, USA) is a GLP-1R and GIPR agonist consisting of 39 amino acids and a fatty diacid moiety that allows binding to albumin. It was approved in 2022 for once-weekly subcutaneous administration [124]. It contains two non-coded amino acid residues, namely α-aminobutyric acid at positions 2 and 13, and a C-20 fatty diacid residue coupled to the lysine at position 20 by a spacer consisting of L-γ-glutamic acid and two 8-amino-3,6-dioxaoctanoic acids [125,126]. Substituting the C-terminal segment of GIP with that of exendin-4 increases the affinity for the GLP-1R. Additionally, the tyrosine residue at position 1 and the fatty acid moiety affect signal transduction at the GLP-1R. Therefore, tirzepatide represents a GLP-1R-biased agonist that shows less potency for β-arrestin recruitment and activation of the GRK2 pathway, and consequently for receptor desensitization [126].

**Table 1:** Overview of physicochemical properties and structure modifications of approved GLP-1 analogs.

Peptide	MW	pI	DPP-4 Susceptibility	Additional Modifications to Peptide Backbone (linker)	Product (Approval Year)	Half-Life	Approved Dosages	Refs.
**GLP-1 (7–36) amide**	3297.6 Da	- ^(1)^	rapid degradation	-	-	1–2 min	-	[54,127]
**Exenatide**	4186.6 Da	4.86	Ala^8^→Gly substitution	-	Byetta^®^ 2005 Bydureon^®^ 2012	2.4 h	s.c.: 5/10 µg	[105,128]
**Lixisenatide**	4858.5 Da	9.5	Ala^8^→Gly substitution	-	Lyximia^®^ 2013 Adlyxin^®^ 2016	3 h	s.c.: 10/20 μg	[110,111]
**Liraglutide**	4813.5 Da	4.9	steric hindrance	Lys^26^: C16 (via γGlu)	Victoza^®^ 2010 Saxenda ^®^ 2015	~13 h	s.c.: 0.6/1.2/1.8 mg; (Saxenda: additional dosages 2.4/3.0 mg)	[43,129]
**Semaglutide**	4113.6 Da	5.4	Ala^8^→Aib substitution	Lys^34^: C18 (via γGlu-2xOEG)	Ozempic^®^ 2017 Rybelsus^®^ 2017	~1 week	s.c.: 0.25/0.5/1/2 mg; oral: R1 3/7/14 mg/R2 1.5/4/9 mg ^(2)^	[46,47,130,131]
**Dulaglutide**	~63 kDa	- ^(1)^	Ala^8^→Gly substitution	Gly^37^: Fc-region of IgG_4_ (via G7SG4SG linker)	Trulicity^®^ 2014	~5 days	s.c.: 0.75/1.5/3/4.5 mg	[120]
**Albiglutide**	~73 kDa	- ^(1)^	Ala^8^→Gly substitution	Arg^36^: albumin	Eperzan^®^ 2014 (withdrawn)	~5 days	s.c.: 30/50 mg	[100]
**Tirzepatide**	4813.5 Da	- ^(1)^	Ala^8^→Aib substitution	Lys^26^: C20 (via γGlu-Ado-Ado linker)	Mounjaro^®^ 2022 Zepbound^®^ 2022	~5 days	s.c.: 2.5/5/7.5/10/12.5/15 mg	[53]

^(1)^ not reliably published. ^(2)^ R1 and R2: oral semaglutide formulations (Rybelsus^®^), more details in Section 1.2.

## 3. GLP-1 Analogs in Clinical Trials for Treatment of Obesity

The main focus of the current clinical research on obesity pharmacotherapies is on the entero-pancreatic, hormone-based drugs, targeting one or more (usually incretin) receptors concomitantly. They are either partial (signal-biased) agonists on GLP-1 receptor or multiple co-agonists, binding to additional targets such as GIP-, PYY peptide-, glucagon-, and amylin receptor. The aim of multipharmacological drugs is to complement the effect of GLP-1 agonism and to enhance the incretin effect, as a similar phenomenon is observed following bariatric surgery. Increasing knowledge about GLP-1 receptor signaling is opening the door to new agonists. The current pipelines are well described in other reviews [13,132], but here, we will highlight some of the most prominent new strategies.

### 3.1. GLP-1- and GIP-Receptor Co-Agonists

Besides the GLP-1 receptor, one additional target is the GIP receptor. The GIPR, same as the GLP-1R, belongs to the glucagon subfamily of class B G-protein coupled receptors (GPCRs) and is present on pancreatic cells (pancreatic α-, β-, δ- cells and pancreatic polypeptide cells), in the central nervous system, blood vessels, immune system, heart, BAT (unlike GLP-1), and bone cells [57,96]. Unlike the GLP-1 pathway, which remains preserved even in the diabetogenic environment, the GIP response is impaired in diabetes [98,99]. It is also interesting to note that weight loss is observed in both pharmacological combinations: GLP-1- and GIP- receptor agonism, as is the case tirzepatide, but also in combination with a GIP receptor antagonism, which is case in AMG 133 [96].

AMG 133 or Maridebart Cafraglutide, developed by company Amgen Amgen (Amgen, Thousand Oaks, CA, USA), is an optimized drug conjugate containing two GLP-1 analogs coupled to human monoclonal antibody against the GIP receptor. It has recently successfully completed phase 2 clinical trials for obesity (NCT05669599) and has proceeded to Phase 3 for once-monthly subcutaneous administration [133,134].

The exact mechanism of weight loss for both combinations is still unclear, but there are several hypotheses. For tirzepatide, three concurrent mechanisms are provided as explanation. Firstly, it is believed that weight loss is based on the biased signaling on GLP-1 receptor towards diminished receptor desensitization. Secondly, the central antiemetic effect of GIP receptor agonism is believed to increase the tolerable dose of GLP-1 component. Lastly, the fatty acid component in tirzepatide is a biochemical tool that could enhance brain delivery and exert higher central appetite-suppressing effect driven by both, GLP-1- and GIP- receptor agonism [99]. On the other hand, the success of GIPR antagonist/GLP-1 agonist combination origins on the theory that GIP receptor agonists induce receptor internalization upon chronic activation and, therefore, function in a similar manner as GIPR antagonists [99,135,136]. However, there are opposing theories suggesting also that the exposure to an antagonist re-sensitizes the GIP receptors, resulting in the increased number of receptors on the cell surface and in restoration of the GIP response [137].

### 3.2. GLP-1/Glucagon Receptors Co-Agonists (GLP-1R/GCGR Co-Agonists)

GLP-1/glucagon co-agonism has also a synergistic effect in the weight loss. Due to the paradoxical catabolic-anabolic actions of glucagon, a combination with a GLP-1 analog manages to achieve a negative energy balance. Centrally, both glucagon and GLP-1 promote satiety, mainly through hypothalamus-vagus-liver axis, but glucagon increases energy expenditure additionally. The increased energy expenditure seems to be result of increased thermogenesis and of various catabolic effects with a subsequent counter-regulatory anabolic responses trying to restore the exhausted metabolites (e. g. hepatic gluconeogenesis). Glucagon also exerts an insulinotropic effect on β-cells in the postprandial state. The appetite-suppressing effect of GLP-1 alone seems to be largely independent of energy expenditure, while antihyperglycemic effects of GLP-1 can effectively counteract the hyperglycemic potential of glucagon. This could provide a plausible explanation for the strong synergistic potential of GLP-1R/GCGR co-agonists and their subsequent success in clinical trials [138,139]. Some of the dual GLP-1R/GCGR agonists, such as Survodutide (Boehringer Ingelheim, Ingelheim am Rhein, Germany) and Efinopegdutide (Merck & Co., Kenilworth, NJ, USA), have already obtained the FDA fast-track designation for treatment of MASH (Survodutide: LiverageTM programme, NCT06632444; Efinopegdutide: NCT05877547) due to their high efficiency in liver fat reduction. In addition to the benefits on liver, GLP-1R/GCGR co-agonists showed a significant improvement of the HbA1c value in diabetes patients [132]. Survodutide has also advanced to phase 3 clinical studies for treatment of obesity (SYNCHRONIZE™ programme). In addition, the phase 3 studies on survodutide are evaluating alternative subcutaneous formulations (NCT07221591) as well as its cardiovascular safety (NCT06077864).

### 3.3. GLP-1/GIP/Glucagon Receptors Co-Agonists (GLP-1R/GIPR/GCGR Co-Agonists)

The GLP-1R/GIPR/GCGR co-agonists are also currently under investigation for their capacity in treatment of T2DM, obesity, and MASH. Retatrutide (Eli Lilly and Company, Indianapolis, IN, USA) is currently in phase 3 of clinical trials (TRIUMPH programme), where its antiobesity effect is being compared with placebo (NCT06383390 and NCT05929066) and with tirzepatide (NCT06662383). Several other ongoing clinical trials are investigating the effect of retatrutide in patients with obesity and other comorbidities such as diabetes, cardiovascular disease, osteoarthritis of the knee, chronic kidney disease (CKD), and chronic low back pain. Another GLP-1R/GIPR/GCGR triple agonists, Efocipegtrutide (HM15211, Hanmi Pharmaceutical, Seoul, South Korea), has completed phase 1 for treatment of obesity (NCT03374241) and is currently in phase 2 for treatment of MASH (NCT04505436).

### 3.4. Other Combinations with GLP-1R Agonists

Further combinations that focus on the synergistic effect with GLP-1 include those with peptide YY (PYY) and amylin analogs, which both promote appetite suppression in the central nervous system. Amylin is a peptide hormone co-secreted with insulin from the pancreatic ß-cells. Its analog cagrilintide is being tested in combination with semaglutide (Cagrisema, Novo Nordisk, Bagsværd, Denmark: semaglutide 2.4 mg/cagrilintide 2.4 mg), which is currently in Phase 3 clinical trials. The resulted weight loss of 17.1% after 20 weeks is significantly higher than the weight loss of 9.5% for the same time period with same dose of semaglutide alone [140].

Peptide YY1-36 is an enteric hormone co-secreted from intestinal L-cells together with GLP-1 and oxyntomodulin. After being cleaved by DPP-4 to PYY3-36, it activates preferentially the Y2 and Y5 receptors in arcuate nucleus of hypothalamus and has a satiating effect due to the Y2 agonism [141]. It was observed that the postprandial concentrations of PYY and GLP-1 increase 10- to 20-fold following the RYGB. This may explain the mechanism of reduced food intake after the surgery, as it was shown that the concomitant inhibition of both GLP-1 and PYY3-36 pathway had an opposite effect, namely increasing the food intake after RYGB [142]. The GLP-1/PYY combination seems promising, as the combination of PYY analog NNC0165-1875 (Novo Nordisk, Bagsværd, Denmark) together with semaglutide (2.4 mg) have already completed the phase 2 clinical trials. However, the project has been terminated without specifying the reason [143].

Bimagrumab is a monoclonal antibody which blocks activin type II receptor and is also being tested in combinations with GLP-1 agonists. It promotes muscle hypertrophy by blocking the atrophic effect of the endogenous ligands, such as activin and myostatin [144]. It is in phase 2 clinical studies for treatment of sarcopenia (NCT02333331). The effects of bimagrumab are, however, not only restricted to myoblasts, but they expand further to adipocytes, concretely to BAT. For instance, lineage tracing show that BAT, unlike WAT, shares common progenitor cells with skeletal muscle tissue. In addition, myostatin is proven to be a potent inhibitor of brown adipocyte differentiation [145]. Bimagrumab, as a myostatin inhibitor, increases the energy expenditure and promotes the lean muscle mass growth even under conditions of a negative energy balance [146]. The combinations of bimagrumab with both semaglutide and tirzepatide are currently being investigated in clinical trials sponsored by Eli Lilly (Eli Lilly and Company, Indianapolis, IN, USA). The phase 2 clinical trial evaluating the combination of semaglutide and bimagrumab (NCT05616013) reported results after 48 weeks of treatment, showing that 63.9% of participants achieved a body weight reduction greater than 15%. Results from the 72-week analysis are still pending. A similar study is underway to assess the combination of bimagrumab and tirzepatide for the treatment of obesity in patients without T2DM (NCT06643728). A parallel trial in patients with T2DM (NCT06901349) was also initiated but has since been withdrawn for strategic reasons by the sponsor.

### 3.5. Small-Molecule Drugs

The term “small-molecule drugs” refers generally to chemically synthesized compounds with a low molecular weight (usually under 500 Da) [147]. The synthetically designed, small-molecule GLP-1 analogs could be a prominent alternative to the peptide drugs, primarily due to the economic reasons.

Orforglipron (LY3502970; Eli Lilly and Company, Indianapolis, IN, USA) is currently being evaluated in several phase 3 clinical studies for the treatment of obesity, each focusing on distinct aspects of disease management. These include investigations into its effects on obesity-related comorbidities (NCT06672939), obstructive sleep apnea (NCT06649045), weight maintenance (NCT06584916), hypertension in obese patients (NCT06948422), obesity in patients with T2DM (NCT06972459), osteoarthritis (NCT07153471), urinary incontinence (NCT07202884), and its efficacy and safety in pediatric participants aged 6–17 years (NCT06672549).

Pfizer had initiated phase 1 and phase 2 clinical trials with another small-molecule GLP-1 receptor agonist, danuglipron (Pfizer Inc., New York, NY, USA). However, during dose-optimization studies (NCT06568731 and NCT06567327), a single asymptomatic participant experienced a potential case of drug-induced liver injury, which resolved upon discontinuation of danuglipron. Following a comprehensive review of data from previous clinical studies, the company decided to discontinue the development of danuglipron. Although the phase 2 study evaluating the combination of danuglipron with the small-molecule GIPR antagonist PF-07976016 was also terminated, Pfizer announced its intention to continue developing PF-07976016 independently. Results from the ongoing phase 2 study in adults with obesity (NCT06717425) are expected in December 2025.

The company Genentech (Genentech, South San Francisco, CA, USA), which is part of Roche group, announced after the acquisition of Carmot Therapeutics (Berkeley, CA, USA) the positive results of phase 1 clinical trials for CT-996 (NCT05814107) [148]. The CT-996 is the same as orforglipron and danuglipron, a small-molecule GLP-1 analog designed to be a biased GLP-1 receptor agonist that preferentially activates cAMP signaling over β-arrestin recruitment [149]. Of note, with this acquisition, Roche also obtained access to portfolio of two other small-molecule drugs: CT-388 and CT-868, which are both GLP-1/GIP receptor co-agonists [150]. Roche (Basel, Switzerland) has recently initiated phase 2 studies on RO7795081 (CT-996) in patients with obesity (NCT07081958) and patients with T2DM (NCT07112872).

The surge of newly initiated clinical studies, together with the intensifying efforts of pharmaceutical companies to keep pace with the rapidly expanding obesity market, underscores the value of small-molecule approaches in overcoming many of the challenges associated with oral peptide administration. However, the question remains to what extent they are specific for target receptors, and what the consequences of potential off-targeting might be [151]. Therefore, the ongoing clinical trials will seek to provide the answers.

### 3.6. Peptides vs. Small-Molecule Drugs

To summarize the current approaches for obesity treatment based on GLP-1 analogs, when it comes to the drug design, there are two main strategies:

(1) Multiple receptors targeting: This strategy involves the peptide drugs which are binding two or more (usually incretin) GPCRs. The rational peptide design and the conserved key residues within binding pockets of GPCRs both enable that a created peptide molecule or peptide conjugate bind specifically to multiple targets at once [152]. Since all of the abovementioned incretin mimetics are agonists to GPCRs, this kind of poly-pharmacology will be very attractive for their future drug development [81].
(2) Biased signaling: Some GLP-1-biased agonists mentioned above, such as orforglipron and danuglipron, activate a biased signal cascade, in which GPCR binds preferentially or almost exclusively to G-protein instead to another associated effector protein, in this case, concretely with the ß-arrestin 2. In this manner, the GLP-1 receptor internalization can be avoided. This concept offers the possibility of selecting the signaling pathway, which will have an increased therapeutic effect, while the undesired downstream signal cascades and consequent side-effects could be minimalized [81]. However, it is important to note that biased signaling can be achieved with peptide analogs as well, and that small-molecule drugs potentially show less receptor specificity than peptide analogs [153].

## 4. Challenges and Strategies to Overcome the Physiological Barriers in Oral Delivery of GLP-1 and GLP-1 Analogs

The late success of GLP-1 analogs and co-agonists demonstrates that we are rapidly approaching treatment efficacy comparable to bariatric surgery [13]. In some publications, this is even termed as “medical bypass”, suggesting that the clinical effects of bariatric surgery are attempted to be replicated [25]. The wide range of emerging drug candidates herewith enables the more tailored treatments, which optimize the individual management of obesity and its associated comorbidities [13]. The route and frequency of administration play a relevant role here, since obesity necessitates chronic treatment. Injections are generally considered to be inconvenient and tend to reduce patient compliance. In addition, subcutaneous injection of GLP-1 analogs has been associated with adverse effects at the injection site, including allergic reactions (pruritus, erythema, and urticaria), the formation of injection-site nodules, and altered skin sensation [154]. Therefore, the oral drug delivery is of the highest priority [155]. This faces, however, numerous obstacles.

The enzymatic degradation of peptides occurs at the three stages: in the oral cavity with amylases, in the stomach with pepsin and cathepsin, and in the small intestine with trypsin and chymotrypsin. Furthermore, peptides are susceptible to pH-mediated hydrolysis in the gastrointestinal tract, enzymatic degradation in the liver (CYP enzymes), and rapid renal clearance [156,157]. Overcoming biological barriers is an additional and also important challenge: the mucus layer is the first line of barrier due to its negative charge and viscosity which can hinder the penetration of peptide drugs. The epithelial barrier represents the subsequent challenge, as peptides are unable to cross the epithelium (transcellular transport) or tight junctions (paracellular transport) by passive diffusion, due to their size and hydrophilicity. All of the aforementioned challenges still significantly limit oral bioavailability; consequently, most peptide therapeutics continue to be administered by intravenous or subcutaneous routes [156].

The current strategies addressing these issues range from structural modifications (e.g., peptide cyclization, D-amino acid substitution, cationization, pegylation, addition of fatty acid chains), to formulation improvements using permeation enhancers, enteric coatings, enzyme inhibitors, polymers for controlled drug release, micro-/nanocarriers, targeted drug delivery, and physical devices [156]. The novel strategies such as highly specific and potent bacterial enzymes, namely, mucin-specific proteases (mucinases) and phospholipases, are also currently being explored due to their ability to enhance mucus diffusivity and membrane permeability [158]. Table 2 provides a summary of these strategies and representative examples described within the scope of this review.

However, with the exception of cyclosporin, the bioavailability of the majority of peptide drugs approved for oral administration is less than 1%. Moreover, only approximately 15% of the investigational peptide drugs currently undergoing phase 3 clinical trials use the oral administration route [156].

The only GLP-1 analog drug currently available on the market in an oral formulation is Rybelsus^®^ (Novo Nordisk), with its main excipient N-acetylated amino acid derivative of salicylic acid designated as SNAC (see Section 5.1). Although safety concerns about SNAC are currently more theoretical, the risk of potentially increased absorption of noxious agents, immunogenic reactions, or altered gut microbiome requires an extensive postmarketing surveillance. The extremely low bioavailability of Rybelsus (1–2%, [47]) is the additional problem and it underlines the need for more efficient and innovative approaches.

However, there are not many studies focusing on the oral formulation of GLP-1 or its analogs. As mentioned earlier, although many pharmaceutical companies are competing in the rapidly growing obesity-drug market, when it comes to oral formulations they typically focus on small-molecule (biased) GLP-1 analogs. Novo Nordisk remains the only company with an approved oral peptide formulation of a GLP-1 analog (Rybelsus^®^), and it continues to explore and optimize SNAC-based delivery technologies. A few other companies have also entered this field, attempting to develop alternative oral peptide delivery systems. For instance, Eli Lilly has initiated the development of an oral peptide formulation with the GLP-1R/GIPR co-agonist LY3493269 (for more details, see Section 5.3). 

A notable line of ongoing research explores the oral delivery of GLP-1 analogs—specifically, liraglutide, semaglutide, and tirzepatide—using the proprietary DehydraTECH™ technology developed by company Lexaria Bioscience (Calgary, AB, Canada). In this approach, the peptide is mixed with triglycerides, deposited onto a substrate such as starch, and subsequently dried. By adjusting the ratio of long-chain to medium-chain fatty acids, the formulation can be optimized to favor either hepatic or lymphatic uptake following intestinal absorption. The company has reported reduced adverse events and improved brain delivery in preclinical and early human studies; however, these findings have not yet been published in the peer-reviewed literature. Currently, a phase 1 clinical trial evaluating an oral DehydraTECH™-semaglutide and tirzepatide formulation is registered (NCT06648031), and results are pending [159,160,161,162].

Following the overview of recent trends and general strategies for oral peptide delivery (including the summary presented in Table 2), we proceed to a more detailed discussion of specific studies and experimental formulations. In particular, the section on micro- and nanocarriers for oral delivery reviewed numerous preclinical investigations primarily conducted in the context of type 2 diabetes. These studies were selected due to their relevance to formulation development, with the outcomes related to delivery efficiency, such as bioavailability and glycemic control, serving as proxies to inform potential applications in obesity management.

## 5. Permeation Enhancers for the Oral Delivery of GLP-1 and Its Analogs

The issue of low gastrointestinal absorption and the consequent low bioavailability of peptides can be addressed by the use of permeation enhancers (PEs) [163,164]. Briefly, PEs represent a versatile class of substances, comprising, for example, medium-chain fatty acids (MCFAs), fatty acid salts, bioadhesive polymers, surfactants, bile salts, and chelating agents [165,166]. These substances are capable of transiently perturbing the intestinal epithelial membrane, thus ameliorating the permeation of macromolecules, while exhibiting minimal local and systemic toxicity [166]. Some of these substances, such as SNAC or certain MCFA, are already acknowledged by the FDA as generally regarded as safe (GRAS) ingredients, which is of great relevance for the development of oral peptide-based pharmaceuticals and the corresponding approval procedures [164]. Depending on their mode of action, the PEs can support different permeation routes (e.g., paracellular or transcellular), and the different PEs can thus be synergistically combined to further increase the permeation rate of the drug across the gastrointestinal barrier [167]. In general, the absorption-enhancing effect of PEs might be affected by a complex network of various parameters including drug residence time, drug concentration, the ratio of PE to drug, environmental pH, and the drug (peptide) itself [165,168].

### 5.1. SNAC (Sodium N-[8-(2-Hydroxybenzoyl)amino]caprylate)

In September 2019, as the FDA and EMA approved the first oral dosage form of semaglutide (Rybelsus^®^), SNAC was brought into the public eye as a gastrointestinal PE. Here, the delivery of semaglutide was achieved by a tablet that remains and completely erodes in the lower stomach region [164]. The SNAC was already granted GRAS status in 2015, when it was used as PE in the first oral formulation of vitamin B12 designed to reach the efficacy of an injection (Eligen B12™, Emisphere Technologies Inc., Roseland, NJ, USA) [169]. It is evident that this helped introducing SNAC as drug excipient in Rybelsus^®^ years later. Novo Nordisk also acquired Emisphere in 2020 to, as the company announced, further develop Eligen SNAC technologies in its current and future drug pipeline assets for biologic medicines [170,171].

Acting as a local buffer, SNAC, a synthetic N-acylated amino acid derivative of salicylic acid, increases pH in the microenvironment, and so it protects the semaglutide from acidic degradation by gastric enzymes. The absorption-enhancing mechanisms of SNAC are also based on its formation of non-covalent complexes with semaglutide, leading to the augmented lipophilicity of the peptide and consequent facilitated transmembrane transport. Moreover, it also facilitates the peptide absorption by fluidizing the biomembranes without affecting the tight junctions and epithelial integrity of mucosa, allowing the peptide to predominantly follow the passive transcellular transport. Finally, SNAC also promotes the monomerization of semaglutide, thus preventing its oligomerization. The oligomerization is an unfavorable process that leads to an increased dynamic size and molecular weight of the drug. This, in turn, has a detrimental effect on the rate of absorption [165,168,169,172].

Chen et al. described a new delivery system for semaglutide that combines physical (microneedle) and non-physical (PE) modes of delivery in a capsule device. Here, they used SNAC as PE. The device was tested in Yorkshire swines as a large animal model. It was demonstrated that the self-triggered microneedle system provides improved tissue interaction and prolonged fixation to the gastric mucosa. This is crucial for the permeation-enhancing activity of SNAC and the resulting local delivery of semaglutide [173,174].

### 5.2. Chitosan

Another example of an “old hand” PE is chitosan [175]. Based on its remarkable permeation-enhancing properties due to its charge, chitosan has emerged as an interesting substance for oral peptide delivery [176,177]. The positive charges of glucosamine residues in chitosan biopolymers under mildly acidic conditions promote electrostatic interactions with the negatively charged mucus layer reducing mucus clearance. Furthermore, the positively charged amino groups can interact with negatively charged integrin α_v_β_3_ on the apical membrane of the intestinal mucosa. This initiates integrin clustering along the cell border, activating the downstream signaling cascade that transiently disrupts tight junction integrity [165,178,179].

For oral delivery of antidiabetic drugs (e.g., GLP-1 and its analogs), various nanoparticle-based formulations have been combined with chitosan and this enhanced the drug uptake and the therapeutic efficiency due to its physicochemical properties [163,180,181,182]. An overview about this nanoparticle-based delivery for GLP-1 peptides is given in the chapter about nanostructured drug delivery systems (see Section 6.4 for more details).

### 5.3. Medium-Chain Fatty Acids (MCFAs)

To develop oral peptide formulations, MCFAs, such as sodium caprylate (C8) and sodium caprate (C10), are studied in depth as PEs with surfactant-like actions. Both fatty acids are FDA-approved food additives, which makes them desirable PEs due to relatively low regulatory hurdles. So far, Rybelsus^®^ and Mycapssa^®^ (Amryt Pharma, Boston, MA, USA) are the only marketed oral peptide-based drugs utilizing PE-based absorption processes. While Rybelsus^®^ tablets use SNAC as PE for semaglutide, Mycapssa^®^ uses C8 in the oral octreotide formulation of a lipidic enteric-coated capsule [164,166].

In detail, C8 is an eight-carbon saturated fatty acid with a molecular weight of 166.19 g/mol and a pKa of 5.19, while C10 is a sodium salt of a saturated fatty acid composed of 10 carbons with a molecular weight of 194.25 g/mol and a pKa value of 4.95 [164]. With respect to the absorption-enhancing effects of fatty acids, the most widely accepted mechanisms for C8 and C10 include the modulation of tight junctions (paracellular) and the perturbation and fluidization of cell membranes (transcellular). Up to now, their absorption-enhancing pathways remain unclear. A possible explanation combines multiple mechanisms depending on the physicochemical properties of PEs, the physiological conditions (cell type and pH) and the concentration of PE and drug at the absorption site [164,165,183,184].

In 2022, Tran et al. (Eli Lilly, Lilly Research) demonstrated that amphiphilic C10 was superior to other tested PEs (e.g., SNAC) in increasing oral absorption of a fatty acid acylated GLP-1R/GCGR co-agonist peptide (4.5 kDa) in a rat jejunal closed loop model [185]. A subsequent study of Tran et al. published in 2023 evaluated the intestinal absorption of GLP-1R/GIPR co-agonist (here termed as LY) and it compared the effects of C10 as permeation enhancer in combination with two different peptide drugs. For comparison, they used C10 in combination with semaglutide and with a GLP-1/GIP analog (here also termed as LY). The data clearly revealed higher oral absorption of the GLP-1/GIP analog in rats compared to semaglutide. This effect was explained by enhanced proteolytic stability of the GLP-1R/GIPR co-agonist and the more pronounced monomeric state of the GLP-1R/GIPR co-agonist in solution. The group also investigated the uptake mechanisms in vivo in rats and minipigs. It was showed that C10 promotes both para- and transcellular pathways of the peptide uptake [186].

The studies on C10 as permeation enhancer continued and the company (Eli Lilly) posted in January 2025 the results of phase 1 clinical studies, where the safety and tolerability of the subcutaneously and intravenously administrated LY3493269 (NCT04178733), as well as the orally given drug (Part 1A: NCT04498390; Part 1B: NCT05794243) were evaluated. Of note, LY3493269 is a GLP-1R/GIPR co-agonist and could be a similar or even the same drug as previously mentioned GLP-1/GIP analog. The study part 1B was terminated by sponsor as enough data was obtained from study part 1A. The orally administered drug was co-administered with C10 in various doses. The drug was administered in doses ranging from 8 mg to 48 mg in a test capsule together with C10 that was co-administered in a separate capsule in one of the two dosages: 250 and 500 mg. The latter (1B) study was designed to have 3 arms and to compare two different test capsules (capsule test 1 and 2, each 4 mg peptide), both co-administered with 280 mg C10. The third arm or the reference group was a tablet (4 mg drug), co-administered with 300 mg SNAC. Of note, a previous another study (NCT04682106) on the same peptide tested up to 600 mg SNAC. The results indicated that the AUC of test capsule 2 was approximately 30% relative to that of the reference tablet formulation. Moreover, the results from recent preclinical studies on monkeys which compared erodible tablets for gastric delivery containing different PEs (C10, SNAC, and 5-CNAC). This study highlighted that the influence of the dissolution profile in combination with a particular PEs on the peptide bioavailability. The study confirmed that both SNAC and C10 are suitable PEs for gastric delivery of the investigated peptide (LY, a GLP-1/GIP analog). The absolute bioavailability for the particular peptide was around 4% in both cases. Of note, the reference formulation containing semaglutide and SNAC showed lower bioavailability [187]. In January 2025, Tran et al. also published a study on mucoadhesive tablets designed for gastric delivery a GIP/GLP-1 analog peptide. The peptide was co-formulated with SNAC in a formulation layer that was surrounded on one or on both sides with mucoadhesive sodium alginate layer. Although the gastric retention was successful, it did not result in increased bioavailability. This can be explained by slower dissolution rate of the peptide in vivo due to the swelling sodium alginate polymer [188].

An interesting study was performed by Niu et al. [167] (Novo Nordisk): they combined the two PEs, SNAC, and C10 in one tablet formulation. Permeability tests were performed in a gastric organoids-based cell model. The combination of SNAC and C10 was superior to either SNAC or C10 alone. Further optimization of tablet formulation was performed by adjusting certain parameters such as PE amount, relative ratio between C10 and SNAC, and the peptide dose. The oral formulation for the GLP-1 analog was tested in beagle dogs, in which the combination of SNAC and C10 was again superior to either SNAC or C10 alone with respect to the bioavailability results. Finally, oral administration was performed in humans, where the combination of SNAC and C10 displayed similar bioavailability compared to the formulation with SNAC only. These results suggested translatability challenges of the permeation enhancement capability of C10 in the stomach between dogs and humans differing in physiology, gastric pH environment as well as concentration of C10 in the gastric lumen. To exploit the full potential of oral peptide therapeutics, this study highlights that more clinically relevant studies are of utmost importance in bridging the gap between preclinical research and clinical application.

### 5.4. Ionic Liquids

Ionic liquids (ILs) represent an alternative permeation-enhancing approach regarding oral peptide delivery development. In brief, ILs represent a class of organic salts being liquid at temperatures below 100 °C. Their low melting point is attained due to bulky and asymmetric organic cations and organic/inorganic anions being present in the structure of ILs. This anion–cation salt structure of ILs shapes their special physicochemical properties including non-volatility, high thermal, and chemical stability, as well as solubility improvement of a wide range of insoluble substances and good water miscibility [189,190,191]. Based on their highly tunable physicochemical properties, ILs allow specific design for delivery systems to fulfill drug-dependent requirements and optimizations. For example, ILs are capable of enhancing solubility of poorly soluble drugs, improving the stability and permeability of drugs in the intestinal mucus as well as promoting penetration across the intestinal epithelial cell barrier [192,193].

Among ILs, choline and geranic acid (CAGE) is an auspicious delivery vehicle for oral drug applications [194]. Banjeree et al. described a highly effective oral delivery system for insulin by incorporating CAGE administered in enterically coated capsules to non-diabetic rats via oral gavage [195]. The study of Agatemor et al. reported beneficial effects of CAGE in subcutaneous GLP-1 delivery [196]. Another approach focused on the preparation of imidazole-based ionic liquids that were functionalized to silica nanoparticles for oral drug delivery of insulin. By integrating ILs into nanoparticles, a controlled and prolonged release profile can be achieved [197]. The described drug delivery approaches might serve as an inspiration to use ILs for future developmental strategies in oral peptide delivery, including GLP-1 applications.

Another interesting aspect of CAGE is reported in the study of Nurunnabi et al. They discussed a body weight reducing effect of orally administered CAGE by decreasing fat absorption through the intestine and concomitantly, providing a feeling of satiety in rats [198]. These observations might provoke the idea of a combinatorial treatment with CAGE and GLP-1 for obesity. Further, recently published data of Rebollo et al. described a salcaprozate-based IL formulation, namely CHONAC for choline salcaprozate. In this work, they have developed this CHONAC IL-based oral formulation for gastric delivery of a GLP-1 analog [199].

Although a lot of beneficial effects of ILs for (oral) drug delivery are reported, it is worth keeping in mind that the permeation-enhancing properties of ILs might correlate with toxicity events. Therefore, a rational design of ILs to guarantee high safety and performance standards is of utmost importance [190,193].

To conclude this section, the use of PEs is a common strategy to improve absorption and bioavailability of oral GLP-1 peptides. PEs with different mechanisms of actions can be combined in formulation development followed by a synergistic enhancement of peptide absorption. PEs should be simple and easy to manufacture while possessing a high safety profile as well as favorable absorption-enhancing effects. The “one-size-fits-all” approach may not be effective for oral peptide formulations because it is a complex interplay between various physiological and physicochemical properties of the tested organism and the applied PEs, respectively [165,185]. 

## 6. Micro- and Nanocarriers for Oral Delivery of GLP-1 and Its Analogs

As reported in the referenced studies [200,201,202,203], the oral relative bioavailability of pure liraglutide and exenatide is nearly 0%, which highlights the importance of an effective drug delivery system. One of the most promising approaches to improve this is the development of nanoparticulate transport systems. The ideal nanoparticles (NPs) for the oral delivery of peptides and proteins should be biocompatible and biodegradable; should have a size below 300 nm; and should be in a spherical shape. These characteristics have been shown to enhance the internalization by gastrointestinal epithelial membranes. Different aspects about the surface charge have to be taken into account. Positive surface charge promotes mucus and cell adhesion due to electrostatic attraction, leading to retention of the delivery system at the site of absorption and increasing the oral bioavailability. A neutral surface charge, on the other hand, minimizes electrostatic interactions with the negatively charged mucus, thereby facilitating the passage through it. In addition, it prolongs the blood circulation time of the nanocarriers, leading to sustained release effects. Nanoparticles with negative charge inhibit closer contact with the intestinal epithelium due to electrostatic repulsion, hindering drug absorption, but can enhance lymphatic uptake. Regarding the stability of nanoparticulate drug delivery systems, strong negative or positive surface charge is favorable to keep the drug delivery system dispersed by electrostatic repulsion of the particles [204,205,206,207,208].

### 6.1. Active Targeting Mechanisms in Oral Delivery via Micro- and Nanocarriers

The main function of the nanocarrier is to protect the active substance from degradation by intestinal fluids and enzymes; however, it can also serve for the purpose of sustained drug release. This is especially relevant for achieving a practical dosing pattern. Furthermore, the surface modifications can be employed for one or more of the following purposes: (1) to provide the means of guidance, (2) to increase the cellular uptake, and (3) if possible, to facilitate navigation of the nanocarrier within the cell and protect it from lysosomal degradation. In other words, the ligands on the surface of nanocarriers can affect not just the manner of uptake into the cell, but also the subsequent transportation route and fate of the nanocarrier. Nevertheless, the primary challenge in the oral delivery of peptides is still overcoming the intestinal mucosa. In this regard, there is potential for targeting of several receptors in the gastrointestinal mucosa.

#### 6.1.1. FcRn Targeting

Currently, the most exploited receptor for active targeting in purpose of oral delivery is the neonatal Fc receptor. In mammals, in addition to transplacental transport in the third trimester, the FcRn has an endogenous function in the infant’s intestine after birth. This involves the transfer of IgG from breast milk across the intestinal mucosa into the systemic circulation [113,209]. Contrary to its expression in rodents, its expression in humans does not decrease with age and the receptor is further present in adulthood in the epithelia of small intestine and colon. Moreover, the receptor is found in other polarized epithelial cells, including those in the lung mucosa, vascular endothelium, blood–brain barrier, as well as in kidney, liver, and haematopoietic cells.

The specific receptor-ligand binding kinetics provide explanation firstly for transport of IgG and albumin across the cell in both directions, and secondly for protection of both ligands from intracellular degradation. The both endogenous ligands of FcRn, namely the Fc-region of IgG antibody and the albumin, can bind to FcRn simultaneously and independently of each other. The binding is for both ligands strictly pH-controlled, with a strong bond present only in an acidic environment (pH < 6.5). In the physiological milieu (pH 7.4), neither IgG nor albumin binds to FcRn [113]. In this way, the FcRn has a significant role in the homeostasis of IgG and albumin. In the acidic environment in the intestine, the ligands can bind to receptor present on the apical side of enterocytes, resulting in the receptor-mediated endocytosis. Thereafter, the ligands bound to the receptor ends up in the endosome compartment where they are sorted out and excepted from the lysosomal degradation. The presence of the receptor on the intracellular vesicle can further guide the vesicle to the basolateral side, where upon reaching the neutral pH, the ligands will be released in the extracellular space of the lamina propria. Additionally, at acidic pH, the uptake via fluid-phase pinocytosis at a neutral pH can also take place. In a similar manner, the acidic milieu in the endosomal compartment will again rescue the ligands from the lysosomal degradation and the presence of FcRn will guide the transport across the cell. Thereafter, in a neutral milieu, the ligand will be released. In this manner, the ligands can be delivered intact to the systemic circulation [113,114,209]. Because of this, the targeting of the FcRn receptor is of special interest for the design of nanocarriers intended for oral delivery of peptide drugs across the intestinal membranes [208].

#### 6.1.2. Transferrin Receptor Targeting

Transferrin (Tf) is an abundantly present serum protein responsible for safe iron transport in a redox-inactive form to supply the growing cells within the body [210,211]. It is highly expressed in human intestinal epithelia, and it is resistant to proteolytic enzymes. Consequently, transferrin is regarded as a useful asset for the surface optimization of nanoparticles and the development of fusion protein conjugates [211]. The binding of iron-loaded transferrin to its receptor (TfR) leads to the receptor-mediated endocytosis. Thereafter, the iron is dissociated from Tf-TfR complex in the acidic endosomal compartment, and it binds to other carrier proteins, while the complex is transported to the cell surface, circumventing the lysosomal degradation [114,210]. Consequently, the conjugation of the transferrin to the peptide drug or the nanocarrier can be leveraged for its escape from the intracellular degradation.

#### 6.1.3. Targeting Goblet Cells

CSK peptide (CSKSSDYQC) was discovered by introducing a random phage-display library into intestine of rats to screen for the peptide sequences able to induce transcytosis over mucosal epithelia [212]. The goblet cells, mucus producing cells representing the second largest cell group in intestinal epithelia, were identified as the gateway for CSK peptide [212,213]. The CSK peptide was first time introduced onto the surface of nanoparticles in trimethyl chitosan chloride (TMC)-based nanoparticles for oral delivery of insulin [214].

#### 6.1.4. Other Targets

Targeting of intestinal receptors for an improved oral delivery of peptide-loaded nanocarriers has been a prominent approach in former decades. Although the previously discussed receptors occurred in a number of studies on oral delivery of GLP-1 and its analogs, several other potential targets emerge as well in this context in the literature. Hyaluronic acid (HA), for instance, has multiple functions in oral peptide delivery. In addition to its expression in cancer cells, the primary receptor of HA (CD44) is also expressed in the intestinal mucosa, where HA has been shown to induce receptor-mediated endocytosis. Beyond that, HA also shows mucoadhesive and protective properties in gastric environment, which is useful for the oral delivery of peptides [215].

As many other transporters of water-soluble vitamins, the folate transporter (proton-coupled folate transporter PCFT, also termed as SLC46A1) is also abundant in intestinal mucosa [216,217]. Interestingly, there have been evidences that folate plasma levels are increased in diabetic patients, which could suggest a higher expression of folate-transporters (PCFT) on the intestinal mucosa. Moreover, the upregulated expression has been already demonstrated in diabetic rats [218]. This could be of advantage for oral delivery of antidiabetic peptide drugs using the nanoparticles with folic acid on their surface [217]. Analogly, another vitamin having potential in intestinal targeting is biotin; however, it seems that here, the current research on GLP-1 analogs for oral delivery remained only on the biotin-PEG-GLP-1 conjugates. Still, the conjugates were able to enhance the intestinal absorption and prolong the half-life of GLP-1 [219,220].

The subsequent chapters will provide a comprehensive overview of the developments in the field of micro- and nanosystems for the oral delivery of GLP-1 and its analogs over the past 25 years, which are also summarized in Table 3. The majority of studies concentrate on the general pharmacokinetics of these drug delivery systems and the treatment of diabetes. Nevertheless, a robust correlation exists between diabetes and obesity, and the findings of these studies are equally pertinent in the context of obesity.

### 6.2. Microparticles of Different Types

Going back in history, the first published work about small scale carrier systems for GLP-1 and its analogs focused on microparticles (MPs) rather than on nanoparticles. It was in the year 2000, when Joseph et al. encapsulated a DPP-4-resistant analog of GLP-1 (D-ala2-GLP-1) in microspheres consisting of poly(lactide-co-glycolide)-COOH (PLGA-COOH) and olive oil (Table 1). The -COOH end group of PLGA was meant to prolong the gastrointestinal passage time and olive oil to enhance the release rate. To deliver therapeutic concentrations of D-ala2-GLP-1 to normal CD1 and diabetic db/db mice by the discussed microspheres, a very high dosage of 250 µg/mouse was used. The study showed that the microparticles lower the glycemic response to orally given glucose significantly compared to pure D-ala2-GLP-1, which lost its activity within 4 h. An extended effect over at least 6 h was achieved by the orally administered microencapsulated D-ala2-GLP-1 [221].

Another type of microparticles was developed by Zhang et al. in 2014 [222]. Exenatide was encapsulated in cross-linked alginate-hyaluronate microspheres, which keep compact in acidic conditions but swell in alkaline medium. Two hours after oral administration, a glucose lowering effect was measured, which lasted for a moderately prolonged period of another 4 h. The relative bioavailability was calculated to be around 10%, meaning that the 10-fold dosing was needed for the oral exenatide microspheres (500 µg exenatide/kg) to achieve a similar effect as the subcutaneously administered drug (50 µg/kg).

One year later, Soudry et al. prepared self-assembled exenatide nanoparticles made of bovine serum albumin (BSA) and dextran, which can be cross-linked via its reactive OH-groups and protect the delivery system from degradation. To further enhance the stability of these primary nanoparticles, reduce the drug release but enhance its absorption in the intestine, they were embedded in gastro-resistant microparticles, consisting of hydroxypropyl methylcellulose (HPMC) and Eudragit^®^ L100-55 (Evonik Operations GmbH, Darmstadt, Germany), which promote the passage through the enterocytes. Drug loading and encapsulation efficiency of the particles stayed rather low. After internalization of the microparticles into enterocytes, they were transported via the lymphatic pathway to the systemic blood circulation as dextran is augmenting the lymphatic uptake. By this, the first pass effect was circumvented and the relative bioavailability of oral microparticles (dosing: 165 µg exenatide/kg) compared to s.c. injection of the product Byetta^®^ and free exenatide solution (dosing: 65 µg/kg) reached stunning 77% and 89%, respectively [204]. Pharmacodynamical investigations in diabetic mice revealed that the formulation (100 µg exenatide/kg twice daily) significantly improved different glycemic parameters to a similar extent as the s.c. exenatide solution (40 µg/kg twice daily) and stopped weight gain [200].

In 2021, Ren et al. had the idea to load exenatide-PLGA nanoparticles into yeast cell wall particles resulting in an irregularly shaped nano-in-micro carrier with adequate sustained release properties. Yeast cell wall particles are porous carriers targeting intestinal phagocytic cells. Their implementation enhanced the gastrointestinal stability of exenatide and improved the blood glucose lowering effect significantly over pure exenatide-PLGA nanoparticles [223].

### 6.3. PLA and PLGA Nanoparticles

Polylactic acid (PLA) and poly lactic-co-glycolic acid (PLGA) are FDA and EMA approved biodegradable polymers which are widely used for the preparation of drug delivery systems for proteins and peptides. They show a high biocompatibility, good stability, and the physical properties of the polymer can be adjusted to achieve sustained release [267].

In 2013, Wang et al. developed the first exendin-4 encapsulating PLGA nanoparticles. These were coated with positively charged, mucoadhesive chitosan, which enhanced their transport through membranes and cellular uptake compared to the free drug and the uncoated particles [224]. One year later, GLP-1 loaded PLGA nanoparticles were compared according to their in vitro permeability with solid lipid nanoparticles (SLNs) and porous silicon (PSi) nanoparticles in a study of Araujo et al. As the described carrier systems all exhibited negative charge due to the primary materials used, they have been coated with chitosan and demonstrated a stronger contact with cells afterwards, as already described by Wang et al. [224]. Coated PLGA nanoparticles, which were the only ones avoiding the release at pH 1.2, showed the best sustained release properties, followed by PSi nanoparticles. The SLNs caused a burst and sole release of about 40% at low pH in the simulated stomach. The sustained release properties of the PLGA nanoparticles were also proven in permeability studies, where they showed the lowest amounts of GLP-1 permeating across intestinal cell monolayers in 3 h [206].

Subsequently, the same working group around Santos and Sarmento studied the PLGA-chitosan and PSi-chitosan nanoparticles in more detail and added the cell penetrating peptide (CPP) polyarginine R9 in order to improve transcellular transport. CPP are small, polycationic peptides which enhance the drug transport across the endothelial membrane and its oral bioavailability without damaging the cells or provoking a cellular response. Furthermore, these nanoparticles were coated with an enteric resistant polymer, to protect the drug and the carrier system from the acidic environment in the stomach, and were loaded with a DPP4-inhibitor to reduce enzymatic degradation of GLP-1. For these modifications, a microfluidic flow-focusing glass device was used. The final size of the spherical shaped particles was pretty large with values around 60 µm. Only insignificant amounts of GLP-1 were released at acidic conditions. After changing the pH to 6.8, a burst release was observed for the PSi nanoparticles followed by nearly no further release, while the PLGA nanoparticles demonstrated a constant sustained release profile. This can be explained by the different binding of GLP-1, which is loaded into the pores of the PSi nanoparticles through physical adsorption, whereas it is encapsulated inside the polymer matrix of the PLGA nanoparticles, resulting in a longer release time. The integration of the CPP into the nanosystem led to an intensification of the cell-particle interactions [225].

In a further study, in vivo tests on diabetic rats have been performed by Araujo et al. for the PLGA nanoparticles. Two hours after oral administration of the system (200 µg GLP-1/kg) the blood glucose levels decreased while the plasmatic insulin levels increased significantly compared to the control group. Blood glucose stayed low for further 6 h. The combination of GLP-1 and DPP4-inhibitor in the nanoparticles resulted in enhanced hypoglycemic effects over the system without DPP4-inhibitor due to the reduced enzymatic degradation of the drug [226].

The working group around Sun K. coupled PLGA with polyethylene glycol (PEG) to circumvent the enzymatic degradation and prolong the circulation time of exenatide loaded PLGA nanoparticles. The PEG-PLGA nanocarriers were conjugated with Fc-groups as targeting ligand to Fc-receptors (FcRn) on epithelial cells. They achieved a longer residence time in the GI-tract, permeated cells faster and to a greater extent, showing a stronger hypoglycemic effect than unmodified nanoparticles. Additionally, extended hypoglycemic effects by oral administration of the Fc-PEG-PLGA nanoparticles (100 µg exenatide/kg) compared to s.c. injection of exenatide (10 µg exenatide/kg) in diabetic mice were proven [227].

In a further study, the invented nanoparticles were loaded with exenatide complexed with zinc ions to improve the stability of the peptide. Here, the NPs have been decorated with transferrin (Tf) instead of Fc-groups. Tf binds to Tf-receptors on enterocytes, thereby the intestinal permeability and cell uptake of the spherical Tf-PEG-PLGA NPs compared to the non-targeted NPs was enhanced (for more details, see Section 6.1.2). The receptor-mediated uptake was proven by a competitive receptor binding of the targeted NPs and free Tf. The relative bioavailability compared to s.c. injection of exenatide was 6.45% [268]. The same working group loaded exenatide-Zn^2+^ into PEG-PLGA NPs functionalized with low molecular weight protamine (LMWP), which significantly enhanced the penetration of the intestinal epithelium compared to NPs without protamine. A quite high release of nearly 50% of the drug occurred at gastric pH within 2 h. Nevertheless, the oral application of these nanoparticles (100 µg exenatide/kg) to diabetic rats led to a better hypoglycemic effect compared to non-functionalized NPs. The effect lasted for 24 h and a relative bioavailability (vs. s.c. injection of 10 µg exenatide/kg) of 7.44% was achieved [228].

The LMWP-PLGA nanoparticles have also been coupled with dextran (DEX) instead of PEG. Positively charged dextran is a hydrophilic, non-toxic polymer, improving drug encapsulation in nanoparticles and enhancing interactions between NPs and GI tract. However, drug loading and encapsulation efficiency were equal to that of LMWP-PEG-PLGA NPs. LMWP enhanced the affinity of the NPs to epithelial cells and the cell internalization as well as transmembrane transport efficiency. The relative bioavailability after oral administration of the NPs (100 µg exenatide/kg) was 8.4% compared to s.c. injection (10 µg exenatide/kg), with a significant and extended hypoglycemic effect on diabetic mice [229]. The LMWP-DEX-PLGA nanoparticles resulted in a 3% higher relative bioavailability than particles without LMWP but could not nearly reach the relative bioavailability of 89% achieved by the BSA-DEX microparticles developed by Soudry et al. [204].

Furthermore, Song et al. invented a block copolymer, CSKSSDYQC-dextran-PLGA (CSK-DEX-PLGA), and prepared exenatide-zinc loaded nanoparticles out of it. CSK shows a special affinity to goblet cells (see Section 6.1.3 for more details) and has already been used by the same research group four years before for the preparation of exenatide loaded chitosan nanoparticles [239]. The now discussed CSK-DEX-PLGA nanoparticles exhibited enhanced mucus permeation and significantly improved the absorption efficiency in the small intestine compared to DEX-PLGA nanoparticles without CSK. Although a high drug release of 60% occurred at gastric pH within 2 h, a prolonged hypoglycemic response and a relative bioavailability of 9.2% (100 µg exenatide/kg oral vs. 10 µg exenatide/kg s.c.) in diabetic rats after oral administration was measured [213].

Ismail et al. were the first who encapsulated liraglutide in PLGA nanoparticles which achieved a 1.5-fold higher cell permeation than liraglutide solution [230,231]. To provide enteric resistance at low pH, Senduran et al. coated liraglutide loaded PLGA nanoparticles with Eudragit^®^ L30D (Evonik Operations GmbH, Darmstadt, Germany). Gastric resistance and sustained release over 48 h, attributed to the PLGA polymer matrix, were observed. Oral treatment of diabetic mice with the invented nanoparticles (75 µg liraglutide/kg and 150 µg liraglutide/kg, respectively) twice a day over 9 weeks showed a significant decrease in blood sugar levels 5 weeks after treatment start for the higher dosed NPs. Interestingly, the body weight was not influenced during the test period [232].

Polylactic acid (PLA) nanoparticles loaded with liraglutide were investigated by Uhl et al. in 2020 [238]. To overcome the poor mucosal penetration, they coated the NPs with a cyclic, arginine-rich cell penetrating peptide (cyclic R9-CPP), similar to that used by Araujo et al. 5 years before [225]. Cyclic CPPs additionally enhance enzymatical stability. An increased cell binding, previously also reported by Araujo et al., resulting in a prolonged retention time, significantly enhanced uptake and bioavailability of the NPs modified with CPP in comparison to unmodified NPs. The relative bioavailability compared to subcutaneously injected liraglutide was not tested. As liraglutide leaked out of the NPs within a few hours during storage at 2–8 °C, a freeze-dried, stable formulation for long-time storage was prepared.

Combining liraglutide-PLGA nanoparticles with a cholesterol derivative in a microfluidic chip was reported to enhance the oral absorption and unidirectional transcytosis of the intestinal epithelium by Wu et al. Additionally, a phospholipid-PEG was included to shield the NPs and permit mucus penetration. The NPs demonstrated rather high drug loading and encapsulation efficiency as well as sustained release properties over 3 h. Daily oral administration over 28 days led to the effective control of hyperglycemia. The effect on glycemic parameters of these oral NPs in a very high dosage of 5 mg liraglutide/kg was comparable to s.c. injection of free liraglutide (0.2 mg/kg) and partially eliminated fat deposition [233].

To achieve smart oral delivery of liraglutide, the working group around Sun K. developed self-ablating zwitterionic dilauroyl phosphatidylcholine-hemagglutinin-PLGA NPs showing an irregular core-shell structure and compared them to self-assembled nanoparticles. Zwitterionic carrier systems facilitate overcoming the mucus and epithelial barriers. The lipid shell of the self-ablating NPs degrades when the NPs are captured by lysosomes and then the inner core is exposed. Hemagglutinin facilitates the lysosomal escape of the PLGA NPs by disruption of the lysosomal membrane. The self-ablating NPs demonstrated higher encapsulation efficiency and cell uptake, better stability, bioavailability, and hypoglycemic effect than self-assembled nanoparticles. The relative bioavailability of the orally administered NPs compared to s.c. injection of free liraglutide solution reached approximately 10% [234].

In recent years, the research group around Santos and Sarmento returned to the investigation of PLGA nanoparticles for the oral transport of semaglutide. This time they focused on PLGA-PEG NPs targeting the FcRn on enterocytes as the working group around Sun K. did in 2018, but they improved the specificity of the targeting system by the use of a small FcRn-targeted peptide (FcBP2) and an affibody (ZFcRn), respectively, instead of the IgG Fc-fragment. To improve the encapsulation efficiency, semaglutide was ion-paired with the polyamine spermidine before loading into FcBP2- and ZFcRn-NPs. Due to the complexation with spermidine, only 5% of the drug was set free under gastric conditions and no further drug was released in the intestinal medium. Cell interactions of the invented NPs were improved by about two-fold compared to non-targeted NPs. The targeted NPs revealed a distinct and enhanced glucose-lowering effect in a humanized FcRn transgenic mice model after single oral application (rather high dosage of 3 mg semaglutide/kg) in comparison to the oral free drug, but no significant difference was observed compared to the non-targeted NPs. Daily oral administration of the FcBP2- and ZFcRn-NPs for 7 days resulted in a similar to higher glucoregulatory effect compared to the s.c. injection of semaglutide (60 µg/mL). These pharmacodynamic results are contradictory to the aforementioned release test, where no drug was set free in intestinal medium [235,236].

Due to concerns that PEG is not biodegradable under normal in vivo conditions and might accumulate in the body, Zhao et al. proposed using amphoteric sulfobetaine as an alternative for coating PLGA NPs loaded with liraglutide. Sulfobetaine is a biodegradable, hydrophilic, and electrically neutral zwitterion, offering better stealth properties than PEG to protect peptide drugs from degradation. After self-assembling on the surface of PLGA-NPs, sulfobetaine enhances the circulation half-life, mucus penetration, cell affinity, and uptake by shielding the negative charge and hydrophobic properties of PLGA. Indeed, the prepared NPs improved mucus and intestinal permeability as well as cell uptake, and the oral relative bioavailability was increased by 4% compared to the free drug. In comparison to s.c. injected liraglutide (54 µg/kg), the orally administered sulfobetaine PLGA NPs (540 µg liraglutide/kg) achieved a relative bioavailability of 9%. Single and multiple oral doses of these NPs lowered the blood glucose level in diabetic mice significantly and to a similar extent as s.c. injected liraglutide [237,269].

### 6.4. Chitosan Nanoparticles

Chitosan is a cationic, natural polysaccharide with a special ability to adhere to mucus and to transiently open tight junctions of epithelial cells, thereby increasing the paracellular permeation. Because of its safety, stability, bio-adhesiveness, pH-sensitivity, and absorption enhancing effect, it is widely used in pharmaceutics [270]. (Further details are provided in Section 5.2).

In 2011, the first exendin-4 loaded chitosan NPs were prepared by Nguyen et al. These self-assembled NPs, consisting of positively charged chitosan and the negatively charged peptide poly(γ-glutamic acid) (γ-PGA), were freeze-dried and filled into hard gelatin capsules coated with enteric resistant polymer (Eudragit^®^ L100-55, Evonik Operations GmbH, Darmstadt, Germany). After reconstitution the particles showed sizes around 260 nm and a positive surface charge. The capsules, orally administered to diabetic rats, exhibited a prolonged hypoglycemic effect. The relative bioavailability of the oral capsule (300 µg exendin-4/kg) compared to s.c. injection of free exendin-4 (50 µg/kg) was 14% [203].

Two years later, Ahn et al. conjugated low-molecular-weight chitosan and cysteinylated exendin-4 via disulfide bonds, which are cleavable in plasma and GI mucus, containing cysteine-rich glycoproteins, to maintain a maximum of active drug. These conjugates, formed by electrostatic interactions, revealed a relative bioavailability (400 µg/kg orally vs. 50 µg/kg s.c.) of rather low 6.4% [205].

The already previously mentioned research group around Sun K. used the CSK peptide to prepare goblet cell-targeting chitosan nanoparticles encapsulating exenatide. The CSK-chitosan NPs achieved a relative bioavailability (50 µg exenatide/kg orally vs. 5 µg exenatide/kg s.c.) of similarly low 6.56% as it was reported by Ahn et al. [205,239]. These nanoparticles have been further refined by the development of a liraglutide loaded core-shell system consisting of chitosan, CSK, hemagglutinin to enhance lysosomal escape and poly-N-(2-hydroxypropyl)-methacrylamide (pHPMA), a hydrophilic and mucus-inert substance for mucosal delivery of nanocarriers. After the nanocapsules pass the mucus, the pHPMA shell is degraded and the particle core is exposed. CSK mediates the cellular uptake by goblet cells where the nanocapsules are partially destroyed, forming smaller spheres which expose hemagglutinin, helping the nanocapsules to escape lysosomes. The invented core-shell nanoconstruct exhibited higher stability and cellular uptake than all of the control groups. The relative bioavailability of the oral nanocapsules compared to the s.c. injection of free liraglutide was 10.12%, 2.25 times higher than that of oral liraglutide solution [240,241].

In 2023, Jakhar et al. prepared liraglutide chitosan NPs coated with the gastric resistant polymer Eudragit^®^ S100 (Evonik Operations GmbH, Darmstadt, Germany). Sustained release was observed over 2 days. The oral treatment of obese mice for two weeks with 100, 200, and 400 µg liraglutide/kg twice a day significantly reduced body weight compared to treatment with 200 µg free liraglutide/kg administered via oral route [242]. 

### 6.5. Silica Nanoparticles

Inorganic silica nanoparticles offer unique features like chemical stability, safety for pharmaceutical use, and large surface areas, making them ideal for hosting guest molecules of various sizes, shapes, and functions. Porous silicon (PSi) nanoparticles have already been successfully used for the oral administration of biomacromolecules. They present several advantages such as top-down production, high surface-to-volume ratio, adjustable particle and pore size, high drug loading capacity, biodegradability, and biocompatibility. Moreover, effortless surface modification of the PSi, either by physical adsorption or chemical conjugation, make their properties easily tunable. The drug loading is usually a simple process under minimal harsh conditions, avoiding possible degradation of the drugs. The drug is retained inside the mesopores, typically showing pore sizes between 2 and 3 nm, by physical adsorption or electrostatic interactions [180,225,243].

Kaasalainen et al. have studied the GLP-1 loading and desorption of PSi NPs in detail. They concluded that electrostatic interactions between peptide and PSi NPs play a minor role compared to hydrophobic interactions as the highest drug loading was achieved when the pH was close to the isoelectric point of the peptide and its charge was nearly zero. About 14% of the loaded drug was bound irreversible to the NP surface, building a fixed layer that facilitates the desorption of the following peptides bound to the NPs [271]. Reviewing the studies of this chapter revealed that all of the drug loaded silica nanoparticles exhibited rather large sizes between 300 and 750 nm. Additionally, at least half of them showed a rapid and rather low release of 25–40% in the intestinal medium within 5–30 min (Table 3).

The first attempt to load silica nanoparticles with GLP-1 was taken by Qu et al. in 2012 [243]. They prepared a rather simple, pH-sensitive nanosystem consisting of Aerosil^®^ 200 (Evonik Industries AG, Essen, Germany), adsorbed GLP-1, and enteric resistant coating with Eudragit^®^ L100 (Evonik Operations GmbH, Darmstadt, Germany). Nonetheless, about 35% of GLP-1 were released at pH 1.2 within 2 h. A burst release of 80% of the drug within 1 h at pH 7.4 is assumed to be the reason for a quite short increase in its plasma concentration for only 2 h. After oral application of the NPs in a rather high dose (1 mg GLP-1/kg), they demonstrated a significant hypoglycemic effect compared to the orally administered pure drug. A relative bioavailability of 35.67% versus intraperitoneal injection of GLP-1 (0.33 mg/kg) was observed. No relative bioavailability compared to s.c. injection was tested.

As already mentioned in the Section 6.3, Araujo et al. compared GLP-1 loaded PSi nanoparticles with SLNs and PLGA nanoparticles according to their in vitro permeability in 2014/15 [206,225]. Subsequently the same working group around Santos and Sarmento investigated these mucoadhesive PSi nanoparticles, whose pores were loaded with GLP-1, in more detail. The mucoadhesiveness was reached again by surface modification with chitosan. As before, these NPs were embedded in an enteric resistant polymer matrix including a DPP-4 inhibitor by an advanced aerosol flow reactor. Only the cell penetrating peptide was not included this time. The NPs showed controlled drug delivery, enhanced mucoadhesion over NPs without chitosan, and improved intestinal permeability compared to the free drug and NPs without DPP-4 inhibitor. Blood glucose levels were significantly improved in diabetic mice compared to the control groups treated with GLP-1/DPP4 solution or empty NPs [180,244]. Three years later, the research group functionalized the previously described NPs with the Fc-fragment of immunoglobulin to target the Fc-receptor on epithelial cells in the intestine. As described above, the working group around Sun K. did the same for exenatide-PLGA NPs at the same time [227]. The DPP-4 inhibitor was not included this time. For the entrapment in the pH-sensitive polymer, matrix glass capillary microfluidics were used, as already conducted before by Araujo et al. [225]. The drug loading and encapsulation efficiency of the final NPs were rather low. The drug was set free in a pH-responsive, sustained manner. The interaction of the NPs with the intestinal cells was increased due to Fc-receptor targeting, enhancing the GLP-1 absorption compared to free drug and NPs without Fc-fragment [245].

Large pore dendritic silica NPs with a pore size of 10 nm were loaded with exenatide and coated with chitosan by Abeer et al. High drug loading, due to the large and dendritic pore structure of the NPs, was achieved. Functionalizing of the NPs with phosphonate optimized the surface charge and drug loading efficiency [246]. In 2020, Lamson et al. described small (<100 nm), negatively charged silica nanoparticles acting as physiochemical PEs that facilitate the oral delivery of exenatide. Specifically, these nanoparticles work not by moving across the intestinal epithelium as delivery vehicles, but by binding intestinal surface receptors that mediate the opening of tight junctions. Enterically coated exenatide capsules (1 mg/kg) were co-administered with the silica NP suspension (200 mg/kg). Compared to the same dose of subcutaneously administered exenatide, the silica-assisted, orally delivered peptide achieved a 10% relative bioavailability in treated mice [228].

### 6.6. Lipid-Based Nanoformulations and Liposomes

One type of lipid-based nanoparticles are the so-called solid lipid nanoparticles (SLNs), consisting of crystalline lipids and showing a diameter between 50 and 1000 nm. SLNs can be used for controlled drug release and drug targeting, they protect their cargo against degradation, are biocompatible, and can be produced easily in large-scale [272]. In 2014, Araujo et al. made an attempt to prepare GLP-1 loaded oral SLNs comparing them to PLGA and PSi nanoparticles as described in Section 6.3, but they had trouble with the drug release from the SLNs (Table 3) [206]. Nanostructured lipid carriers (NLCs), consisting of crystalline and liquid lipids, surfactants, and water, are the successors of SLNs [273]. Additionally, to the aforementioned advantages of SLNs, NLCs show the ability to stimulate the endogenous GLP-1 secretion from enteroendocrine cells by mimicking the effect of lipid nutrients. Interestingly, the single lipid components alone do not affect the GLP-1 secretion, only the composition of lipids within an NLC is effective [274]. In 2018, Shrestha et al. encapsulated exenatide as well as liraglutide in NLCs. A very high encapsulation efficiency around 90% was reached and about 20% of the drug was released under gastric conditions, whereas 60–100% of the drug was set free by a burst release within 30 min in the intestinal medium. Although in vitro studies proved an enhanced permeation of NLCs compared to free drug solution across intestinal cells in the absence of mucus, in vivo and ex vivo studies revealed that the NLCs became stuck in the mucosa and could not reach the epithelial cells in the intestine. Further modifications to overcome the mucosal barrier were required.

As the developed NLCs additionally showed a GLP-1 secretory effect on enteroendocrine cells, they were promising dual-action oral delivery systems that were worth being further researched [247]. Therefore, the working group changed the carrier system to lipid nanocapsules, containing reverse micelles (RM-LNCs) loaded with exenatide. The drug was entrapped with a remarkable efficiency of 85%. The final RM-LNCs demonstrated gastric resistance and prolonged release in the intestine. The endogenous secretion of GLP-1 was additionally triggered by the empty RM-LNCs with a size of approximately 200 nm. In a former study, it was postulated that smaller sizes do not achieve a GLP-1 secretory effect in in vitro cell tests, but this effect was not observed in human ileal organoids in a present study of the research group [275,276]. The relative bioavailability after oral administration of exenatide-loaded RM-LNCs (500 µg exenatide/kg) compared to s.c. injection of the free drug solution (50 µg/kg) was low, revealing values around 4%. Nevertheless, it was approximately three-fold higher than that of the orally administered exenatide solution (500 µg/kg). The invented nanosystem demonstrated its dual-action of endogenously secreted GLP-1 and intestinal delivered exenatide following acute and chronic treatment of obese/diabetic mice [248].

To further enhance the endogenous GLP-1 secretion and prolong the glycemic effects of the invented nanocapsules, the research group proposed to use PEGylated RM-LNCs loaded with exenatide instead. The improved formulation enabled an administration every second day, showing similar effects to the daily applied formulation and equal effects to the daily applied PEGylated RM-LNCs without exenatide loading in diabetic mice [249]. In 2024, the RM-LNCs were also tested for the delivery of semaglutide. Again, a very high encapsulation efficiency of 90% was reached and a better trend for glucose reduction was observed during a 4-week daily oral treatment than with exenatide RM-LNCs, pure RM-LNCs, and free drug, respectively [250]. Finally, the research group around Sun K. published a study about exenatide-loaded folic acid (FA)-modified RM-LNCs, targeting enterocytes as folate receptors are abundant in gastrointestinal tract. The folic acid was combined with PEG and phospholipids (DSPE) before the inclusion in RM-LNCs. The cell uptake and transcellular transport of these nanocapsules (FA-RM-LNCs) was significantly improved over that of unmodified RM-LNCs, resulting in a 1.28-fold increased bioavailability. The relative bioavailability of the orally given FA-RM-LNCs (100 µg exenatide/kg) compared to s.c. injection (10 µg/kg) reached 7.5%. The hypoglycemic effect of a single oral dose of FA-RM-LNCs was significantly higher than after oral application of exenatide solution and showed prolonged characteristics over the subcutaneously injected drug. After multiple dosages of FA-RM-LNCs over 5 days, blood glucose levels were reduced by approximately 30% while the unmodified RM-LNCs and the subcutaneously injected drug reached reductions about 20% [217].

A further relevant group of nanoparticles made of lipids are self-assembled, spherical phospholipid bilayer vesicles called liposomes. Liposomes are highly biocompatible and biodegradable. Their physical and chemical properties can be easily modified to design effective drug carriers [252]. Exendin-4-liposomes coated with chondroitin sulfate-g-glycocholic acid (GCA) were described by Suzuki et al. GCA is an important part of the human bile salts and was chosen as coating to enhance the intestinal absorption. By these orally applied liposomes (200 µg exendin-4/kg), a comparably high relative bioavailability versus s.c. administration of the drug solution (20 µg exendin-4/kg) of 19.5% was reached. A prolonged release of the drug was proven in the intestine. Unfortunately, gastric release was not tested. The oral application of the exendin-4-liposomes (300 µg/kg daily) over 4 weeks revealed a significant reduction in body weight. At this point, it is important to highlight that this reduction in body weight was even stronger than that achieved by daily s.c. injections of 20 µg/kg free exendin-4 solution [251]. Liraglutide loaded corona cationic liposomes were recently published by Ding et al. Bovine serum albumin (BSA) was adsorbed on the surface, leading to enhanced mucus permeation because of its hydrophilic properties and the nearly neutral surface charge. During the penetration process, the outer shell of the liposomes gets degraded, exposing the cationic core of the liposomes. The liposomes were additionally linked to AT-1002, a hexamer synthetic peptide that increases intestinal permeability by opening the tight junctions between the cells. The liposomes showed prolonged release, good physiological safety, and enhanced absorption via intra- and transcellular transport. The relative bioavailability after intra-jejunal application of the liposomes (540 µg liraglutide/kg) was 9% compared to s.c. injection of the free drug (54 µg/kg), an improvement of 5% over free liraglutide applied into the jejunum. Further studies should focus on enabling oral administration of the formulation [252,253].

Finally, self-emulsifying drug delivery systems (SEDDSs) and nanoemulsions belong to the lipid-based nanoformulations. SEDDSs consist of lipids, surfactants, and co-solvents, which spontaneously form oil-in-water nano- or microemulsions with the gastric fluids after application. They can help to overcome the low oral absorption of peptide drugs by excluding hydrolyzing enzymes from the lipophilic SEDDS droplets, enhance mucus permeation, due to their slippery surface and their good absorption membrane permeating properties, and control drug release [254,255]. Nearly all of the herein reviewed articles about SEDDSs and nanoemulsions demonstrated a comparatively elevated relative bioavailability over other nanosystems.

In 2018, the first paper about exenatide SEDDS was published by Menzel et al. To efficiently incorporate exenatide in the lipid SEDDS droplets, the working group increased the lipophilicity of exenatide by hydrophobic ion pairing with the anionic surfactant sodium docusate (DOC). A 20% (*m*/*v*) dilution of the SEDDS in phosphate buffer pH 6.8 resulted in stable and relatively low droplet sizes around 45 nm. The SEDDS enhanced mucus permeation and reached a relative bioavailability of approximately 15% compared to s.c. injection of exenatide (SEDDS: 150 µg exenatide/rat vs. 20 µg exenatide/rat s.c.) [254]. Two years later, these exenatide-DOC SEDDSs were compared to exenatide SEDDSs ion paired with the stronger anionic sodium n-octadecyl sulfate (SOS). All of the SEDDSs demonstrated sizes below 30 nm. The membrane permeability was enhanced by exenatide-SOS SEDDS 3.5-fold over the exenatide-DOC SEDDS and 6.4-fold over the exenatide solution. Compared with the s.c. injection of the exenatide solution (50 µg/kg), exenatide-SOS SEDDS (300 µg exenatide/kg) achieved a relative bioavailability of notable 19.6% and exenatide-DOC SEDDS 15.2% [255].

The same working group around Bernkop-Schnürch additionally compared hydrophobic ion pairing of exenatide with the cationic surfactant tetraheptylammonium bromide (THA) to exenatide-DOC, respectively. The prepared exenatide-THA and -DOC nanoemulsions exhibited enhanced intestinal membrane permeability and an even higher relative bioavailability of remarkable 28% and 16%, respectively, after oral administration (300 µg exenatide/kg) compared to s.c. injection of the free drug (50 µg/kg). The huge difference in the bioavailability of the described nanoemulsions can be explained by the different surface charge, which was positive for the exenatide-THA NCs and negative for the exenatide-DOC system. The invented drug delivery systems showed droplet sizes below 30 nm [256]. Lin et al. developed an exenatide loaded oil-in-water nanoemulsion with an amazing drug encapsulation efficiency of 96%. The lipid droplets of this system are liquid at 37 °C, semi-liquid at room temperature, and solid at a storage temperature of 4 °C, providing long-term stability. The oil droplets effectively protected exenatide from degradation in the gastrointestinal tract and were expected to possess higher cell surface binding, cellular association, and consequently enhanced cell uptake due to their liquid and deformable properties. The authors postulated an increased intestinal lymphatic uptake due to the negative charge of the droplets and a resultant pancreas targeting. The relative bioavailability after oral application of the nanoemulsion (300 µg exenatide/kg) compared to s.c. injection of exenatide (50 µg/kg) was quite high (23.8%) and the drug release was prolonged [257].

In recent years, Lu et al. invented reverse micelle-SEDDSs (RM-SEDDSs), with exenatide loaded in the hydrophilic core of phospholipids. The SEDDSs were coated with the natural, anionic polysaccharide hyaluronic acid, leading to enhanced nanoparticle adhesion and cellular uptake. The surface potential of the double-layered nanoparticles stood close to zero and sustained release properties were observed. An enhanced hypoglycemic response over the uncoated NPs for at least 12 h after oral application was proven, but the orally applied dosage was rather high (1 mg/kg) [215].

RM-SEDDSs have also been tested by the research group around Bernkop-Schnürch in 2024, when they studied their potential as carriers for semaglutide in comparison to hydrophobically ion paired SEDDSs. Their results presented a higher lipophilicity for RM, which resulted in a significantly enhanced encapsulation efficiency in RM-SEDDSs, better stability, and prolonged release. Both types of SEDDSs significantly improved cell permeation, demonstrating the beneficial effect of lipophilic complexes for the delivery of semaglutide [277].

### 6.7. Various Special Nanoparticles

Protamine Nanoparticles

Exenatide-Zn^2+^ was combined with deoxycholic acid-low molecular weight protamine to prepare nanocomplexes by Han et al. in 2021 [258]. Low molecular weight protamine is a non-toxic, cationic peptide, that enhances cell penetration and prevents exenatide from intracellular degradation. To target apical sodium-dependent cholic acid transporters in the ileum, hydrophilic, negatively charged glycocholic acid-poly (ethylene glycol)-b-polysialic acid copolymers were introduced in the drug delivery system. Polysialic acid is an endogenous polysaccharide that enhances stability and transport of NPs. The dual cholic acid-functionalized NPs were filled into enteric-coated capsules for their oral administration. In vitro tests in simulated intestinal fluid containing trypsin revealed a high protection of exenatide, loaded into the invented drug delivery system, against degradation. The release profile showed prolonged characteristics, cell uptake and transport were increased and ileum targeting was realized. After oral gavage of the NPs (300 µg exenatide/kg) to diabetic rats, a relative bioavailability of 12.32% compared to s.c. injection of 20 µg exenatide/kg was measured and a distinct loss in body weight was recognized.

Cyclodextrin Nanoparticles

Cyclodextrins have been successfully used as excipients for oral drug delivery systems with low toxicity. Presas et al. have used amphiphilic cyclodextrins, functionalized with cationic groups and PEGylated-phospholipid chains (mPEG-DSPE 2000), to form electrostatic complexes with liraglutide and further self-assemble to NPs, which protect the drug from enzymatic degradation. Finally, a dextran sulphate coating was added to the nanocomplexes. Liraglutide was encapsulated to a striking 100%. After intra-jejunal application of the NPs (200 µg liraglutide/kg), they reduced the glycemic response in rats significantly and to a similar extent as s.c. injection of 200 µg liraglutide/kg. The oral administration of the invented nanocomplexes has not been tested so far [259].

Metal Organic Framework Nanoparticles

Metal organic framework (MOF) NPs, consisting of inorganic clusters bridged by organic ligands, possess a high encapsulation capacity for exendin-4 because of their large pore size and surface area. Zhou et al. used iron MOF nanoparticles equipped with amino groups which allow trapping of negatively charged peptides by electrostatic interactions and provide the opportunity for further modifications. A remarkable encapsulation efficiency of 87% was realized. The working group coated the NPs with a hydrophobic, zwitterionic hydrogel layer to enhance mucus permeation and internalization by epithelial cells. The NPs were loaded together with the effervescent components, citric acid and sodium bicarbonate, into pH-sensitive capsules to protect them from degradation in gastric acid. The capsule shell dissolves in the intestine and CO_2_ bubbles are set free, because of the acid and bicarbonate, which promote the movement of the NPs. Increased mucus permeation and endothelial cell uptake was proven due to the CO_2_ propulsion and the hydrophobic, zwitterionic surface of the NPs. The exendin-4 release from the invented MOF NPs showed a prolonged characteristic in the intestine and a relative pharmacological bioavailability of 17% was reported after their oral application (400 µg exendin-4/kg) to diabetic rats compared to s.c. injection of exendin-4 (80 µg/kg) [260,278].

Zein Nanoparticles

Bao et al. used a special corn protein, called zein, as basic material for the preparation of exenatide embedded nanoparticles. Zein is a prolamin with a high amount of hydrophobic amino acid residues. Therefore, it is not soluble in water but in ethanol and binds other ingredients by hydrophobic interaction. Additionally, phosphatidylcholine was incorporated to enhance the stability of the peptide drug and the transportation over the intestinal epithelium. Cholic acid was used to promote intestinal absorption via bile acid transporters, as performed one year before by Suzuki et al. for liposomes [251]. Casein acted as stabilizer by suppressing hydrophobic aggregation of the NPs and hypromellose phthalate protected exenatide against degradation in the gastric fluids. Two variants of the zein NPs were tested: one was prepared by dispersing all of the single ingredients in zein (DIS NPs) and for the second one, exenatide was complexed to micelles with phosphatidylcholine and cholic acid first (COM NPs) to increase the loading efficiency from 53.6% to 79.7%. Nearly no drug release occurred in the simulated stomach, followed by an initial burst release and subsequent sustained release in the intestinal medium. COM-NPs exhibited better sustained release properties, protection of exenatide against enzymatical digestion, and intestinal absorption than DIS NPs. The achieved relative bioavailability compared to s.c. exenatide injection (60 µg/kg) for DIS NPs and COM NPs (600 µg exenatide/kg) were 5.6% and 10.9%, respectively [261].

Three years later, the same working group proposed similar NPs, replacing phosphatidylcholine and casein by rhamnolipids, surface-active glycolipids that improve aggregation stability by reducing the hydrophobicity of zein NPs. This time, liraglutide complexed with cholic acid was used as incorporated drug and hypromellose phthalate was not contained anymore. Sustained release was observed. Rhamnolipids and cholic acid improved the mucus and transcellular permeation of liraglutide as well as its oral absorption compared to NPs without these excipients. The orally applied nanoparticles, in a high dosage of 4 mg liraglutide/kg, reached a relative pharmacological bioavailability of 9.6% compared to s.c. injection of free liraglutide solution (400 µg/kg) [262].

Another type of zein nanoparticles for the transport of liraglutide was created by Ziebarth et al. The NPs were stabilized by pH-sensitive Eudragit^®^ RS100 (Evonik Operations GmbH, Darmstadt, Germany) and mucoadhesive chitosan. They demonstrated a rather good persistence in gastric and intestinal fluids and prolonged release properties. The orally applied NPs achieved an area under the curve (AUC) of approximately 693 ng*h/mL in rats. Compared to the s.c. injection of free liraglutide, which was applied in the same dosage (200 µg liraglutide/kg) and achieved an AUC of approximately 978 ng*h/mL, a relative bioavailability of impressive 71% can be calculated [182]. As an additional remark, it is important to mention that oral zein NPs have been proven to stimulate the endogenous secretion of GLP-1 by Reboredo et al. even without drug loading, mimicking the effect of protein nutrients [279]. In this respect, a combinatory effect of stimulated endogenous production of GLP-1 and delivered drug is to be expected for zein NPs loaded with GLP-1 or its analogs.

Milk Exosomes

A rather new approach to deliver liraglutide and semaglutide is the encapsulation in milk exosomes. These are extracellular vesicles with sizes about 30–150 nm, low immunogenicity, efficient cargo protection, and stable gastrointestinal properties. The restrictions of milk exosomes are their limited mucus permeation, because of repulsive forces and steric effects, their low drug loading efficiency, and the possible degradation of membrane proteins on their surface, which aid their internalization. To overcome these challenges, Xiao et al. have designed hybrid vesicles with self-adaptive surface properties. Therefore, they fused milk exosomes with liposomes and introduced a highly hydrophilic zwitterionic polymer linked via pH-sensitive hydrazone bonds to phospholipids (DSPE-Hyd-PMPC) during the preparation of liposomes. Due to these modifications, the encapsulation efficiency of semaglutide was enhanced over natural milk exosomes, membrane proteins were preserved, and the mucus was traversed efficiently by the invented delivery system. The drug loading ranged around remarkable 30%. The freeze-dried vesicles were loaded into enteric resistant capsules and bypassed the stomach after oral administration. In the intestine, the zwitterionic hydration layer improved the mucus penetration because of its strong hydrophilicity and neutral charge. Approaching the intestinal cell surface, the hydration layer was detached under the weakly acidic conditions and cationic lipids as well as membrane proteins of the vesicles were revealed, improving cellular uptake and transport, respectively. After oral administration of the vesicles (300 µg semaglutide/kg) a relative bioavailability of 9% was determined in comparison to s.c. injection of the free drug (30 µg/kg). Their blood glucose lowering effect was similar to subcutaneously injected semaglutide for 6 h; within the following 6 h, the hybrid vesicles showed enhanced effects over the s.c. applied drug and significantly intensified hypoglycemic effects compared to orally administered free drug and natural milk exosomes, respectively. Blood lipid-related indicators showed improved values in diabetic rats after oral treatment with the developed hybrid vesicles over 6 weeks, comparable to that of animals treated with s.c. injected semaglutide over the same period of time [263].

Aminoclay Nanoparticles

Aminoclay is a 3-aminopropyl-functionalized magnesium phyllosilicate, which is positively charged and, therefore, provides the ability of electrostatic interactions with negatively charged drugs. It shows a low risk of toxicity and can improve thermal stability of proteins. As aminoclay is facilitating trans- and paracellular transport, it can consequently enhance cell uptake. In 2023, Song et al. published their research on aminoclay liraglutide nanocomplexes prepared by spontaneous self-assembly. After reversing the negative surface charge by citric acid, the NPs were coated with negatively charged, pH-sensitive Eudragit^®^ S100 (Evonik Operations GmbH, Darmstadt, Germany), leading to particle sizes of about 370 nm. The drug entrapment efficiency with a value of >90% was exceptionally high. The drug release at pH 1.2 remained below 30%, whereas there was a rapid and extensive drug release of about 70% observed within 1 h at pH 7.4. The nanocomplexes revealed enhanced cell permeability compared to pure liraglutide. After oral application of the nanocomplexes in a very high dosage of 15 mg liraglutide/kg to diabetic mice for 30 days, significant loss in body weight was observed compared to the oral application of the pure drug in the same dosage. Compared to s.c. injection of liraglutide (0.3 mg/kg), approximately half of the body weight reduction was achieved [264]. The same transport system was additionally tested for the delivery of semaglutide, leading to similar characteristics as the liraglutide aminoclay NPs (Table 3). Only the zeta potential was strongly negative this time and the drug release at pH 7.4 was less pronounced. Cell uptake was enhanced again by the NPs and significant therapeutic effects concerning glycemic control and body weight were measured [265].

Bile Acid Nanoparticles

Bile acid derivatives are potent, biocompatible drug carriers, which boost the uptake and oral bioavailability of pharmaceuticals because of their binding affinity to apical sodium-dependent bile acid transporter and a type of G-protein coupled receptor at the epithelial surface. They influence lipid metabolism and, consequently, are of interest for the treatment of obesity. Kweon et al. have recently developed nanoparticles by electrostatic interactions of liraglutide, taurolithocholate (TLC), and lysine-conjugated deoxycholic acid (LDC). These particles have been stabilized by the addition of PEG and the surfactant Labrasol^®^ (Gattefossé, Saint-Priest, France). The drug encapsulation efficiency reached an outstanding 98%. After high-dosed oral application of the NPs (10 mg liraglutide/kg), they achieved a quite low relative bioavailability of 4% compared to s.c. injection of liraglutide (0.5 mg/kg), which was determined to be 0% for free oral liraglutide (10 mg/kg). The glucose lowering effect in diabetic mice after a high single dose of NPs (10 mg liraglutide/kg) as well as after daily administration for 28 days was equal to or even slightly stronger than that of s.c. applied liraglutide (0.1 mg/kg). Application over 28 days resulted in lower body weight and food intake as the s.c. administered drug [202]. Due to trouble with uncontrolled precipitation during the preparation and low production yield, the formulation had to be changed. Instead of taurolithocholate, the negatively charged bile acid sodium deoxycholate (DC) was used for the ion-pairing with liraglutide and LDC. The non-ionic surfactant n-dodecyl-β-D-maltoside (DDM) replaced Labrasol^®^, leading to the formation of nanomicelles. PEG was not included anymore. The new nanomicelles demonstrated similar characteristics as the former NPs (see Table 3) and aggregation, precipitation as well as oxidative degradation were prevented. The freeze-dried product was filled into enteric resistant capsules. After daily oral administration of the invented nanomicelles (10 mg liraglutide/kg) over a period of 12 weeks, they exhibited a similar glucose-lowering effect as s.c. injected liraglutide (1 mg/kg) and higher body weight reduction at a dose of 5 mg liraglutide/kg. The weight of adipose tissue was significantly decreased in diabetic mice compared to diabetic mice without treatment. Oral treatment with nanomicelles in a dosage of 20 mg liraglutide/kg reduced the adipose tissue weight to a stronger extent than s.c. injected drug. Additionally, the oral nanomicelles and s.c. injected drug reduced the adipocyte size, and superior regulation of lipid metabolism was obtained than without treatment [266].

In summary, several attempts to enhance the oral bioavailability of GLP-1 and its analogs by nanosized drug carriers have been made during the last 25 years, reaching from widely applied PLGA NPs over lipid-based NPs to innovative approaches like the use of milk exosomes as transport systems [263,280]. Main aspects that have to be considered are the protection of the peptide drug against acidic and enzymatic degradation, enhanced mucus adhesion, and permeation as well as cell interaction and cell permeability. Finally, increased cell uptake, unidirectional transport through cells, and storage stability are important features to produce most effective formulations. It can be postulated that most of the tested nanoparticles improved the oral bioavailability of GLP-1 and its analogs compared to the free drug, showing prolonged release and extended effects as well as low cell toxicity. Several studies reported about oral nano delivery systems for GLP-1 and its analogs which reached the same or even better glucoregulatory and weight loss effects than a s.c. injection of drug [200,202,233,236,237,251,259,263,266]. Nevertheless, to the best of our knowledge, none of these nano- or microparticulate systems have been studied in clinical trials by now for oral delivery, much less reached an approval by the FDA or EMA. Reasons are assumed to be financial aspects, lack of the detailed toxicity studies, and of profound proofs for prolonged effects in humans. Finally, upscaling of the manufacturing process, particularly the peptide synthesis as the main bottleneck, is still the primary challenge for manufacturers. The complexity of required facilities along with surging demand underscore the importance of developing new, forward-looking strategies that will align capital spending with future infrastructure needs [281].

## 7. Gene-Based Delivery of GLP-1

In addition to the GLP-1 peptide delivery strategies, the activation of a GLP-1 receptor by the exogenous introduction of GLP-1 nucleic acid is another promising strategy for the treatment of hyperglycemia and obesity [282]. Given that the peptide-based therapeutics require a very frequent dosing regime, as well as higher doses necessary for compensating the short half-life of GLP-1, the use of gene delivery could provide a more stable expression, production, and secretion of GLP-1 [283].

The main action of nucleic acid-based drugs is carried out in the intracellular space; therefore, the nucleic acids must be transported through the cellular, as well as the nuclear membrane. However, a major challenge in the delivery of naked nucleic acids is their instability (they can be easily degraded by nucleases), and they demonstrate poor cellular uptake. Once they have crossed the cellular membrane, the nucleic acids must be released from their carrier in order to exert their effect.

The GLP-1 encoding sequence that is commonly used in gene transfer experiments is the sequence for the activeGLP-1(7–37). The cDNA is composed of a methionine start codon for translation start, a furin peptidase recognition site (RGRR), and of a sequence for a secretory signal peptide, which will target GLP-1 to the constitutive secretory pathway [283,284].

Thus far, numerous delivery systems have been developed for the delivery of GLP-1 cDNA, both viral and non-viral, which can stabilize the nucleic acid and provide efficient transfection while minimizing the off-target effects [285]. As they have proven to be excellent genome-packaging systems, for a long time, viruses have been used as vectors. The most used ones are adenoviruses, adeno-associated viruses, and lentiviruses. Although viral vectors have proven success as delivery systems [286,287,288,289,290], there are still some downsides to their use, such as limited genome packaging size, integration into the host genome, pre-existing immunity, as well as an immunogenic reaction provoked by the viral carrier. To overcome these limitations, synthetic delivery systems have been developed as gene delivery systems. They offer protection from enzymatic degradation, increase the circulation time in the bloodstream, provide tissue and cell specificity, and can improve the cellular uptake. Liposomes, naturally occurring peptides, and synthetic polymers belong to the class of non-viral delivery systems. They are characterized by low immunogenicity, biocompatibility, and high gene-loading capacity. When it comes to the delivery of GLP-1 gene, the following strategies have been developed so far: viral vectors, peptide-antibody constructs, formation of polyplexes using polyethyleneimine (PEI), arginine-based polymers, chitosan-based complexes, protamine-based complexes, and complexes based on protamine and bile salts.

As previously discussed in the scope of this review, a current hot topic regarding the GLP-1 peptide therapeutics is the oral delivery. This trend is also present in the gene delivery field. Nurunnabi et al. achieved oral delivery of GLP-1 plasmid DNA (pDNA) by complexing the nucleic acid using branched polyethyleneimine (bPEI), and coating the surface of the obtained polyplexes with heparin-taurocholic acid [291]. The idea behind using bile acids as a coat arises from the fact that their binding to the bile acid binding protein can facilitate the oral absorption of macromolecules. The coated nanocomplexes were negatively charged (-35 mV) and had a size of 160 nm. Given that the uptake of taurocholic acid-coated nanoparticles is mediated by sodium/taurocholate co-transporting polypeptides (NTCP) and apical sodium bile acid transporters (ASBT), the authors used NTCP-rich HepG2 cells to evaluate the transfection efficiency of the coated polyplexes and demonstrated that the coating improved the cellular uptake and transfection. Next, to assess the intestinal transport, the authors used a Caco-2 transwell system, mimicking the tightly packed intestinal epithelial monolayers. The taurocholic acid coat facilitated the transport of the polyplexes from the apical to the basolateral side of Caco-2 cells. Furthermore, the GLP-1 expression level was evaluated after transfecting intestinal L-cells (NCI-H716), and it was shown that the coated polyplexes resulted in a three-fold higher expression compared to the control. The studies were also conveyed in vivo in Zucker diabetic fatty (ZDF) rats and high-fat diet (HFD) induced diabetic mice, where it was demonstrated that the coated complexes were distributed in the liver and small intestine. The orally administered coated polyplexes expressed GLP-1 in vivo, which led to an increase in insulin secretion and reduced blood glucose to normal levels.

Another approach for GLP-1 gene oral delivery was published by Nie et al. [292]. The goal of the group was to overcome the low transfection efficiency resulting from the entrapment of nucleic acids in the mucus layer and epithelial barrier of the gastrointestinal (GI) tract. Their approach includes the use of GLP-1 DNA complexes with linear polyethylenimine (lPEI), coated with a mixture of a neutral lipid, dipalmitoyl-sn-glycero-3-phosphocholine (DPPC), and 1,2-dimyristoyl-rac-glycero-3-methoxy poly (ethylene glycol)-200 (DMG-PEG), which would render the nanoparticles hydrophilic and electrostatically neutral. The nanoparticles were prepared using a technique for continuous production, called flash nanocomplexation. After coating, the diameter of the nanoparticles ranged between 80 and 100 nm, whereas their charge was -1.5 mV. In vitro, the nanoparticles were tested in 293T cells where they demonstrated satisfactory transfection levels. In vivo, they were tested in Balb/c mice, where the expression of the GLP-1 peptide was observed within 12 h and was maintained for 24 h. Furthermore, the blood glucose level of the murine model was evaluated, and normoglycemia was observed 6 h after dosing. Weight reduction was also observed in the treated animals, which is in line with the effects that GLP-1 has on body weight control.

Hasan et al. [293] addressed the topic of oral delivery of GLP-1 DNA by using an antibody-conjugated protamine as a delivery system. Here, protamine was conjugated to a human IgG-Fc, targeting the (FcRn), known to mediate the transcytosis of IgG across epithelial cells (see Section 6.1.1). The protamine component is the one that condenses GLP-1 DNA and provides stability for the nucleic acid. The hydrodynamic diameter of the complex was in the range of 100–200 nm, with a stable profile throughout various pH values used to simulate the GI tract. Cellular uptake studies conducted in HT-29 cells demonstrated that complexation by protamine and the Fc-conjugation led to a higher transfection efficiency compared to GLP-1 DNA alone. Biodistribution studies in diabetic mice demonstrated that the complexes were specifically accumulated in the small intestine, which is well matched with the distribution of the FcRn. What is more, a gradual decrease in the blood glucose level was observed in diabetic mice treated with the complex, compared to control groups treated with the single components, where no effect was observed. Lastly, although no significant effect was observed in the body weight of the mice, the GLP-1 treated group demonstrated lower food consumption compared to the PBS-treated mice.

The conjugation capability of protamine, and mimicking bile acid physiology was utilized by Shahriar et al. [282] to produce a multimodal delivery system for oral GLP-1 gene therapy. Here, they used protamine sulfate because of its cell penetrating abilities, as well as the protection of GLP-1 DNA from DPP-4. Calcium phosphate was added to the formulation as a transfection enhancer, and taurocholic acid was used to protect the formulation during the transition through the GI tract, as well as to facilitate the absorption through ASBT-mediated endocytosis. The nanoparticles were characterized by a slightly negative charge (−5.1 mV), and an average diameter of 232.22 nm. The ability of taurocholic acid to target the ASBT was tested in MDCK cells, and it was compared to the efficacy of non-targeted nanoparticles. The targeted nanoparticles demonstrated better uptake compared to the non-targeted ones, demonstrating the significance of the interaction between taurocholic acid and the ASBT. The formulation was tested in vivo in diabetic mice, where it exhibited favorable GLP-1 expression, which lead to increased insulin secretion and regulated blood glucose levels in the mice. Furthermore, the data suggest that the formulation had a positive effect on obesity by reducing the body’s endogenous fat content. When compared to the GLP-1-based products already available on the market (semaglutide and liraglutide), the formulation was shown to be more potent. What is more, the authors suggest that one oral dose is equivalent to 20 s.c. injections of insulin.

## 8. Microbiota for GLP-1 Delivery and In Situ Secretion

The human gut represents the digestive and immune organ of the body, in which the colonized gut microbiota form a specific community representing a complex ecosystem of a balanced variety of microorganisms (e.g., bacteria, yeasts, fungi, viruses) and exhibiting a symbiotic relationship with its host [294]. Alterations in gut microbiota composition depend on many factors, such as host genetics and health status, antibiotic use as well as various environmental factors including (ketogenic) diet, sleep, and exercise [295,296,297,298].

As GLP-1 is mainly produced by enteroendocrine L-cells in the gastrointestinal system, the gut microbiome can influence GLP-1 secretion as well as its production and function and thus, playing an important role in human health conditions. Changes in gut microbiota have been correlated with a variety of physiological disorders including a loss of insulin sensitivity, impaired intestinal barrier function, low-grade inflammation as well as lipid accumulation—all conditions that increase the risk of metabolic disease development such as obesity and T2DM [294,296,299].

At this point, special attention can be laid on prebiotics and probiotics in ameliorating obesity and T2DM by positively modulating gut peptide production, in particular, GLP-1 [294,299]. As an example, Zeng et al. mentioned a commercial product (designated as VSL#3) containing a total of eight probiotic strains that leads to an increase in GLP-1 and decrease in BMI in children with non-alcoholic fatty liver disease [294]. It is also worth taking note that the gut microbiota can modulate adipose tissue metabolism and thermogenesis, thus holding a promising approach for the treatment of overweight and obesity by influencing energy metabolism, nutrient absorption, appetite as well as adipose tissue development and function. Further, specific gut microbiota have shown promoting or abolishing effects in browning of WAT, which lead to an increase in energy expenditure and improvement in metabolic health in mice. Therefore, using gut microbiota for an enhanced GLP-1 production has emerged as a potential therapeutic pathway for the treatment of obesity and metabolic disorders [295].

### 8.1. Advanced Microbiome Therapeutics (AMTs)

Advanced microbiome therapeutics (AMTs) represent an innovative in situ strategy for the oral delivery of peptide-based medicines using engineered microbes [300,301]. As summarized in the review by Vazquez-Uribe et al. [300], preclinical data on AMTs in animal models of metabolic disorders have shown promising results. More specifically, two genetically engineered probiotic strains were highlighted as delivery systems for recombinant GLP-1 and exendin-4, using *Escherichia coli* Nissle [302] and *Saccharomyces boulardii* [303], respectively. Remarkably, these plasmid-based AMT interventions lead to a significant decrease in food intake and weight gain in mouse models of obesity.

#### 8.1.1. Challenges for Oral Peptide Delivery with AMTs

AMTs for oral peptide application fulfill a dual function: first, as delivery vehicle and second, as in situ production facility for peptides. However, the gastrointestinal tract possesses several protective mechanisms, including the acidic pH of gastric fluids, enzymatic degradation activity in the digestive system as well as functional barrier within the intestinal epithelium—all of those factors contribute to a harsh environment for peptide therapeutics and thus, impairment of their stability and absorption behavior [163,304]. In fact, the tasks of AMT-based delivery systems include peptide protection from enzymatic degradation followed by intact peptide secretion and enhanced peptide absorption in the gastrointestinal tract, which can be further improved by enteric coating and colonic targeting [163,300].

Probiotic microbes (e.g., *Lactobacillus*, *Bifidobacterium*, *Saccharomyces*, *Escherichia*) have been mainly used as AMTs because they are known for their safety use in humans and their manufacturing feasibility [305,306]. The following technological aspects for AMT development have to be considered: First, robust colonization control of AMTs must be achieved, keeping in mind that the interindividual variation of the human microbiome might lead to considerable variability in the colonization capacity of microbial strains. Second, development of AMTs for oral delivery involves controlling the rate of peptide production as well as the dynamics of AMTs growth and clearance from the body. Reaching the desired peptide concentration in the target area are of utmost importance for desired therapeutic effects. Third, biosafety of the engineered microbiota has to be guaranteed [300].

#### 8.1.2. AMTs for Delivery of GLP-1 and Its Analogs

AMTs have attracted attention for delivering GLP-1 and GLP-1R agonists in metabolic diseases, such as obesity and diabetes. These engineered bacteria have passed extensive assessments in both in vitro [307,308,309] and animal models, involving mice [310,311,312], rats [307], and monkeys [313] showing enhancements in the secretion of insulin and tolerance of glucose. In current works, these bacteria-based modular systems are commonly made of plasmid vectors employing signal peptides to produce and transport peptides or proteins [163].

Lactic acid bacteria (LAB), a kind of Gram-positive bacteria that belongs to the normal flora of gut microbiota of human hosts, represent novel promising oral delivery vehicles for GLP-1 and its analogs. Some LAB strains are able to successfully reach the human gastrointestinal tract and colonize the surface of intestinal epithelial cells in situ while protecting therapeutic drugs from digestive enzymes, showing probiotic effects itself [311,314]. For example, Agarwal et al. constructed a delivery system for native GLP-1 based on recombinant *Lactococcus lactis* (LL-pUBGLP1). Based on in vitro studies, they demonstrated insulinotropic activity on HIT-T15 cells and analyzed the transport behavior across MDCK cell monolayer of secreted GLP-1. Further, the efficacy of the recombinant construct LL-pUBGLP1 after oral administration was tested in ZDF rats [307]. By using recombinant *Lactococcus lactis* strain, another study supported the positive effects of bacterially engineered GLP-1 on insulin and glucose regulation in mice on chow or high-fat diet [310]. Furthermore, *Lactobacillus plantarum* [311,313] and *Lactobacillus paracasei* [309] were chosen as oral vectors and for in situ production of recombinant GLP-1 and exendin-4, respectively. The antidiabetic effect of the microbially produced GLP-1 was confirmed with *Lactobacillus plantarum* WCFS1 in db/db mice and *Lactobacillus plantarum*-pMG36e-GLP-1 in a rhesus monkey model [311,313].

Recently, optogenetically engineered bacteria represent a new strategy as drug delivery systems for in situ host metabolism regulation by combining the advances of synthetic biology with micro-nano technology and microelectronics. As an example, Zhang et al. constructed an optogenetic *Lactococcus lactis* system to promote and control GLP-1 secretion in rats by using a wearable optical device under the remote control of blue light [308]. Another approach investigated the influence on blue-light-responsive *Lactococcus lactis* for GLP-1 delivery to manipulate brain function in Parkinson’s disease in mice [315]. As the gut microbiota and the brain are well connected via the so-called gut–brain axis, manipulation of GLP-1 receptors in areas of the brain might represent a valuable pharmacological strategy to treat obesity by influencing food intake, satiety, and thus, body weight control [316,317]. To address the problem of light penetration, other studies were carried out by using near-infrared light as it is known to exhibit improved tissue penetration properties compared to blue light. Zhang et al. designed a red-light-controlled probiotic system by using *Escherichia coli* Nissle 1917 for exendin-4 delivery under optogenetic control in the murine gut [190].

In 2024, another group presented a lipid-coated design for engineered bacteria protecting probiotics from gastrointestinal environment and improving the survival and retention rate in db/db mice. In detail, GLP-1-expressing plasmids are introduced into *Escherichia coli* Nissle 1917 followed by thin film dispersion method to coat the bacteria with DLPC (Dilauroyl phosphatidylcholine)-based lipid membranes. DSPE-PEG (1,2-distearoyl-sn-glycero-3-phosphoethanolamine-N-[poly(ethylene glycol)]) and cholesterol were used for improving the stability and prolonging the circulation time of the bacteria-based delivery system, representing the in situ factory for GLP-1 production. Furthermore, calcium ions were added during the formation process of bacteria-GLP-1-coated lipid membranes to facilitate the self-assembly of lipid membranes on the surface of *Escherichia coli* [312]. Another group proved the antiobesity effect of genetically modified *Escherichia coli* Nissle 1917 secreting GLP-1 in mice fed with a high-fat diet [302]. In contrast, Hedin et al. used the probiotic yeast *Saccharomyces boulardii* to produce recombinant exendin-4. By combining cold exposure with bacteria-induced release of exendin-4, the results revealed significantly stronger suppression of food intake and body weight gain compared to the outcomes observed at room temperature [303]. Further, long acting GLP-1 (101aGLP-1) was expressed by *Saccharomyces cerevisiae* resulting in declined blood glucose levels in type 2 diabetic mice after oral administration [318].

Overall, AMTs offer a potential solution by providing advantages over traditional drug administration methods and making the manufacturing of therapeutic peptides more affordable. Nevertheless, most developments are still in the preclinical stage, which results in an auspicious challenge by translating the findings from animal models to humans owing to the biological differences between species [295,300].

## 9. Safety Studies and Misuse of GLP-1 Analogs

The majority of adverse events (AEs) reported in clinical studies on GLP-1 receptor agonists and their analogs are typically mild, with gastrointestinal disorders being the most common. The risks associated with incretin-based therapies include cholelithiasis, micronutrient deficiencies, and loss of lean muscle mass [13]. A recently published study on oral semaglutide (Rybelsus^®^ 25 mg, OASIS 4) reported nausea, vomiting, dyspepsia, and gastroesophageal reflux as the most frequently observed adverse effects. Dysesthesia, or altered skin sensation, was reported by approximately 5% of participants, while pulse rate was only slightly higher compared with the placebo group [319].

The prevention of lean muscle loss is currently the focus of several ongoing clinical studies investigating the co-administration of myostatin inhibitors, selective androgen receptor modulators (SARMs), or other pro-anabolic agents to counteract incretin-induced muscle loss (RO7204239 monoclonal antibody, NCT06965413; enobosarm, NCT06282458; pindolol benzoate, NCT07101939). Another research direction aims to address the common adverse effects of nausea and vomiting. Several agents are under clinical investigation for combination therapy with incretins, including dopamine (D_2_) receptor antagonists and neurokinin-1 receptor antagonists (metopimazine mesylate, NCT06500429; tradipitant, NCT06804603). One study on metoclopramide focuses on its use to reduce residual gastric contents in patients receiving incretin therapy prior to surgery, as delayed gastric emptying may increase the risk of pulmonary aspiration during anesthesia. (NCT07100691)

As therapeutic dosages increase, the potential for off-target interactions also rises. More and more reports are raising the concern regarding their effect on mental health, especially their role in the development of suicidal ideation within users [320]. Chen et al. conducted a postmarketing pharmacovigilance study on the correlation of semaglutide and liraglutide use and suicidal ideation/self-injury, to evaluate the real-life scale of this issue. Their study demonstrated that there is still no evidence to suggest a heightened risk of self-injury with the use of GLP-1R agonists. Nevertheless, they suggest that a large scale, more comprehensive prospective investigations are needed in order to have their findings enhanced [321].

The emergence of new and more convenient orally available therapies also raises concerns about their potential for misuse. The high efficacy of GLP-1 receptor agonists in obesity treatment has made them broadly accepted and has garnered a substantial attention from the general public. What is more, the endorsement by high-profile public figures and the on-going media coverage of Ozempic^®^ (semaglutide), as well as other GLP-1 analogs, demonstrate a rising pattern in their off-label use [322,323]. The increasing public interest in the use of GLP-1R agonists, especially their use for cosmetic weight loss in non-obese individuals, is made visible by the ever-increasing online search popularity and social media presence [322,324,325].

GLP-1R agonists, as previously mentioned, exert influence on the brain’s appetite center, which promotes satiety and decreases the need for food consumption. The onset of action is fast, and the observed weight loss varies between 15% to 20% of total body weight for the newer agents, such as semaglutide. This mechanism of action may be the driving force behind a potential misuse of GLP-1 receptor agonists in non-obese individuals, due to societal standards of physical attractiveness [326]. Indeed, this phenomenon of misuse has been facilitated by the ease of acquisition from unauthorized websites, or compounding pharmacies. Moreover, reports for forged semaglutide prescriptions for non-obese and non-diabetic individuals led to an increased level of surveillance for semaglutide, due to misuse worries. A recent pharmacovigilance study conveyed by Chiappini et al. concluded that semaglutide is at highest risk of being abused, misused, and used without a prescription, compared to other GLP-1 analogs. The most vulnerable populations at risk in this case are the eating disorder (ED) populations [327].

Eating disorders (such as anorexia and bulimia nervosa, as well as binge-eating disorders) are characterized by a distorted body image and abnormal eating patterns. As such, individuals with ED are known to abuse or misuse substances for image enhancement, as they can suppress appetite, increase the metabolism, and lead to weight loss (such as amphetamine or illegal drugs). GLP-1R agonists can now be classified as agents used for image enhancement, and thus, can be categorized as substances which can be abused by individuals with ED. However, there are reports about the possible beneficial effects that GLP-1 receptor agonists might exert in individuals with binge eating disorder. It is speculated that the slowed gastric emptying, as well as the effects that GLP-1 receptor agonists have on the brain’s award center, are responsible for decreased binge eating episodes and weight loss in these individuals. A special attention and more in-depth research are crucial when it comes to the misuse of GLP-1 receptor agonists in ED populations, given that oral drug delivery forms of GLP-1 analogs are being developed, and they increase the misuse risk.

With regards to the use of GLP-1 analogs in weight management in a clinical setting, case studies demonstrate that these agents have the potential to worsen restrictive eating patterns due to their influence on appetite regulation. Therefore, it is of great necessity that patients are screened for eating disorders before they are prescribed GLP-1R agonists. Patients ought to be intensively monitored too, given the potential of GLP-1 to lead to extreme weight loss.

## 10. Conclusions

The prevalence of obesity is rising rapidly, having more than doubled since 1990 among adults [3]. Although several drugs have been approved for the treatment of obesity, this may not be sufficient. The most effective treatments, particularly concerning long-term results, remain surgical interventions [25]. Moreover, obesity management requires a holistic approach that can support individual treatment plans [25,328]. To achieve this, specialized physicians and various groups of medical care personnel need to be involved. In addition, a broad range of drugs should be available to clinicians to facilitate personalized therapy regimes [13]. However, there are still a number of drugs currently on the market, where the side effects may potentially outweigh the benefits (e.g., amphetamine derivatives and other centrally active drugs) [28,29].

GLP-1R agonists have been approved for two decades for the treatment of T2DM and are considered an established therapeutic class for its management. Over that time, the newly approved GLP-1R agonists have consistently demonstrated improved pharmacokinetic properties. With the recent approvals of semaglutide and liraglutide for the treatment of obesity, the GLP-1R agonists have once again gained attention within the research and clinical communities [43,46]. As was demonstrated in clinical studies, the dosage of the GLP-1 analogs tends to be increased in the treatment of obesity, reflecting the differences in physiology between obese and diabetic patients. Unlike those with diabetes, obese individuals maintain both, GLP-1 and GIP responses. This creates opportunities to leverage more pathways for the treatment of obesity (as in case of tirzepatide) [51,53]. The exploration of multireceptor targeting (e.g., GLP-1R, GIPR, GCGR) and biased signaling at the GLP-1R has led to the development of novel peptides [81,152,153]. Concomitantly, efforts to develop small-molecule drugs with comparable receptor binding kinetics have also begun [148,149,150].

However, the main route of administration of GLP-1 analogs is still by s.c. injection. This may pose a challenge to patient compliance; therefore, the development of oral drug delivery systems has become a priority. The first approved oral formulation of a GLP-1 analog (Rybelsus^®^) relies primarily on the use of a permeation enhancer (SNAC) [28,47]. The resulting bioavailability is, however, still very low (1–2%) [47]. Moreover, only a limited number of oral peptide formulations for GLP-1 or its analogs have progressed to clinical evaluation. Although numerous companies are actively developing incretin-based therapies, only a few have advanced sufficiently to establish clinically validated peptide-drug formulations. The success of Novo Nordisk’s Rybelsus has once again demonstrated that simpler formulations—those containing a single, well-chosen permeation enhancer such as SNAC—are often the most viable from an industrial perspective, primarily due to their scalability and manufacturing feasibility. A similar trend is observed in Eli Lilly’s programs, which focus on another permeation enhancer, C10, as revealed in their comparative preclinical and clinical studies [186,187,188].

As research expands, possibilities multiply—from designing more stable peptide structures to optimizing co-formulation strategies. Studies (e.g., with C10) have shown that dissolution profiles and drug-release kinetics significantly influence the performance of permeation enhancers [187]. Their efficacy, in turn, depends on complex interrelations between parameters such as drug-to-enhancer ratios, solubility, and charge properties. Despite this intricate network of factors, the most effective solutions may not need to be the most complex. The demand for higher peptide loading per oral dosage unit remains a major formulation challenge, while the scalability of peptide synthesis continues to be a significant bottleneck in manufacturing. These hurdles underscore not only the technological but also the financial importance of innovative investment strategies and advanced production infrastructures [281].

Beyond peptide delivery, alternative strategies, such as gene-based delivery systems and advanced microbiome therapeutics, are being explored. While promising, these approaches face many of the same hurdles as oral peptide delivery, with even greater complexity in terms of scalability. Their potential advantage lies in achieving simplified dosing regimens, although this benefit remains to be conclusively demonstrated. Moreover, unique challenges persist, such as mitigating immunogenicity risks in viral vectors and managing colonization dynamics in microbiome-based systems.

In conclusion, GLP-1 analogs continue to hold remarkable promise for metabolic and weight management therapies. Their rational use guided by healthcare professionals and informed by robust risk–benefit evaluation can ensure both safety and efficacy. The advent of more efficient oral delivery platforms could dramatically enhance patient adherence and therapeutic outcomes. Moreover, innovative (nano-)formulations that improve delivery efficiency may reduce the demand for high-purity drug quantities, offering economic and environmental advantages. Ultimately, the future of oral GLP-1 therapy will not hinge on the most complex technologies—but on the smartest simplifications that turn scientific potential into scalable reality.

## Figures and Tables

**Table 2:** Overview of strategies for oral peptide delivery, with a focus on the approaches and representative examples related to the GLP-1 analogs discussed in this review.

Barrier/Challenge	Strategy	Mechanism	Examples/Substances
Hydrolytic degradation	Enteric protection	Prevent peptide degradation in stomach	HPMC, Eudragit^®^ L100-55, L30D, S100, Hypromellose phthalate, gastro-resistant capsules
Hydrolytic and enzymatic degradation	Peptide complexation, embedding	Hydrophobic interactions and hydrogen bonds, local pH buffering	SNAC, ionic liquids, cyclodextrins, SEDDS, reverse micelles, NLCs
	Peptide ion pairing	Electrostatic interactions, increased hydrophobicity, and protease resistance	Sodium docusate, octadecyl sulfate, tetraheptylamonium bromide, spermidine, polyamines
	Sustained release formulations	‑ Controlled peptide release via diffusion, swelling, or polymer degradation ‑ Peptide release through cleavage of peptide–polymer conjugate	‑ PLGA, cross-linked alginate-hyaluronate microspheres ‑ Cysteinylated peptide-chitosan conjugates
Enzymatic degradation	Amino acid modifications within peptide	Protease-resistant structures (D-amino acids, substitution at cleavage site)	D-amino acids, other non-native amino acids (α-aminoisobutyric acid)
	Peptide–ion complexation, cyclization	Increased rigidity and protease resistance, conformational stabilization	Zn-exenatide complexation,
	Steric hindrance	Conjugation to other, larger structures	PEG, fatty acids
	Co-formulation with enzyme inhibitors	DPP-4 inhibitors	Sitagliptin, saxagliptin
Mucus & Tissue barriers	Muco-adhesion	Increase residence time of carrier at mucosa	Chitosan, HA
	Physical fixation	Maintain carrier at mucosal surface	Microneedles
	Modulation on the surface of drug carrier	Reduce adhesive (hydrophobic or electrostatic) interactions with mucus	Chitosan, ionic liquids, zwitterionic polymers, BSA, polysialic acid, dextran sulfate
	‑ Hydration of mucus, reducing viscosity ‑ Degradation within mucus	‑ Effervescent components to enhance transport through mucus, enzymatic degradation of mucus ‑ Gradual degradation of the carrier’s outer layer in mucus, exposing the inner layer (often more hydrophobic) near the mucosal surface.	‑ Effervescent excipients, mucinases ‑ milk exosomes with pH-sensitive cleavable coatings DSPE-Hyd-PMPC, pHPMA
Transcellular uptake	Increasing peptide lipophilicity, monomer stabilization (hydrotropic effect)	Peptide complexation to increase its hydrophobicity, prevent its aggregation (protecting hydrophobic regions)	SNAC, fatty acid conjugates, reversed micelles, ion pairing (spermidine, polyamines), ionic liquids, co-solvents
	Membrane fluidization	Facilitation of passive diffusion, disrupting the regular packing of the lipid bilayer, membrane perturbation	Medium-chain fatty acids, SNAC, ionic liquids, co-solvents, specific phospholipases
Paracellular uptake	Tight junction modulation	Transiently opening of tight-junctions for paracellular transport	Chitosan, silica NPs, AT-1002 hexamer peptide
Targeted uptake	Receptor-mediated endocytosis	Enhancing uptake via specific receptors (receptor-mediated endocytosis)	FcRn, bile salts, transferrin, FA, CSK peptide, HA, dextrane, polysialic acid
	Lymphatic uptake	Mostly size- and lipophilicity-dependent, absorption via lipid transport pathways (chylomicrons), via Peyer’s- Patches or M-cells	Dextran, lipid-based carriers, lipid-complexed peptides, yeast cell wall particles
Intracellular stability	Lysosomal escape, basolateral targeting	Circumventing lysosomal degradation, enabling unidirectional transport	Cholesterol, hemagglutinin, CPPs, protamine, cationic aminoclay particles
	Stealth properties	Reduce phagocytosis (and indirect intracellular degradation)	PEG, sulfobetaine, polysialic acid
endogenous GLP-1 release	Carrier-based stimulation	Trigger endogenous GLP-1 release	NLCs (nanostructured lipid carriers)

**Table 3:** Summary of micro- and nanoparticles for the delivery of GLP-1 or its analogs (ZP = zeta potential, DL = drug loading, EE = encapsulation efficiency, BAR = relative bioavailability (p.o. vs. s.c.), PBAR = relative pharmacological bioavailability (p.o. vs. s.c.), → medium was exchanged to pH ≥ 6 subsequently, * referring to glycemic response, ° referring to blood concentration of drug).

Type	Drug	Additional Excipients	Size	Shape	ZP	DL	EE	Oral BA_R_	Oral PBA_R_	Release pH 1–2	Release pH ≥ 6	Oral Single Dose	Oral Single Dose Effect Duration	Refs.
MPs	DPP4-resistant GLP-1 analog	PLGA-COOH, Olive oil	1 µm	-	-	-	-	-	-	no	Yes, 3–5 h after intake	≈10 mg/kg	min. 6 h *	[221]
	Exenatide	Alginate, Hyaluronate	4.1 µm	spherical	-	3%	71%	10%	-	≈15% in 2 h, ^→^	≈85% in 4 h	500 µg/kg	4 h °	[222]
	Exenatide	BSA, Dextran, HPMC, Eudragit^®^L 100-55	1–15 µm	-	-	0.15%	40%	89%	-	≈20% in 2 h	≈80% in 4 h	165 µg/kg	5.5 h °	[200,204]
	Exenatide-PLGA	Yeast cell wall particles	3.9 µm	irregular	+2.1 mV	1.3%	49%	-	14%	≈12.5% in 2 h	≈17.5% in 8 h	300 µg/kg	min. 12 h *	[223]
PLGA NPs	Exendin-4	Chitosan	≈300 nm	-	+17–+25 mV	-	-	-	-	-	-	-	min. 3 h after i.duo. admin.	[224]
	GLP-1	Chitosan	198 nm	spherical	+17.5 mV	0.15%	60%	-	-	<3% in 2 h, ^→^	21% in 4 h	-	-	[206]
	GLP-1	Chitosan, polyarginine R9, HPMC acetate succinate, DPP4-inhibitor	277 nm before HPMC coating, afterw. ≈60 µm	spherical	+21.6 mV before HPMC coating	0.07% before HPMC coating	60% before HPMC coating	-	-	<2% in 2 h	≈30% in 6 h	200 µg/kg	min. 6 h *	[225,226]
	Exenatide	PEG-PLGA, human polyclonal IgG Fc	140 nm	-	−4.9 mV	-	84%	-	-	-	-	100 µg/kg	min. 12–24 h *	[227]
	Exenatide-zinc	PEG-PLGA, LMWP	114 nm	spherical	+2.5 mV	6%	71%	7%	-	40–50% in 2 h, ^→^	≈40% in 10 h	100 µg/kg	min. 24 h *	[228]
	Exenatide-zinc	Dextran, LMWP	141 nm	spherical	+0.16 mV	5%	69%	8%	-	-	-	100 µg/kg	min. 24 h °	[229]
	Exenatide-zinc	CSK-DEX-PLGA	136 nm	spherical	+0.15 mV	5%	72%	9%	-	60% in 2 h, ^→^	10–15% in 10 h	100 µg/kg	min. 24 h °	[213]
	Liraglutide	-	189 nm	spherical	−27 mV	-	52%	-	-	14% in 2 h, ^→^	4% in 4 h	-	-	[230,231]
	Liraglutide	Eudragit^®^L30D	159 nm	spherical	+15 mV	7%	75%	-	-	<5% in 2 h	≈70% in 6 h + ≈15% in 42 h	-	-	[232]
	Liraglutide	25-hydroxy-cholesterol, phospholipid-PEG	165 nm	spherical	-	23%	83%	-	-	≈10% in 2 h, ^→^	≈30% in 6 h	5 mg/kg	min. 3 h *	[233]
PLGA NPs	Liraglutide	Dilauroyl phosphatidyl-choline, Hemagglutinin	152 nm	irregular	−2 mV	-	75%	10%	-	20–30% in 2 h, ^→^	50–60% in 12 h	540 µg/kg	min. 24 h *°	[234]
	Semaglutide	PEG-PLGA, Spermidine, FcBP_2_, Z_FcRn_	≈170 nm	spherical	≈−2 to −3 mV	2–3%	≈60%	-	-	5% in 2 h, ^→^	no	3 mg/kg	min. 8 h *	[235,236]
	Liraglutide	Sulfobetaine	87 nm	spherical	−4 mV	10%	77%	9%	-	≈15% in 2 h, ^→^	≈65% in 22 h	540 µg/kg	min. 24 h *°	[237]
PLA NPs	Liraglutide	Cyclic R9-CPP	≈250–350 nm	-	−1 to −2 mV	-	70–77%	-	-	-	-	n.a.	min. 24 h °	[238]
Chitosan NPs	Exendin-4	γ-PGA, enteric resistant capsule	261 nm	-	+34 mV	15%	61%	14%	-	no	yes	300 µg/kg	min. 12 h *	[203]
	Exendin-4-cys	-	120 nm	spherical	+37 mV	-	-	6%	-	-	-	400 µg/kg	min. 3–6 h *°	[205]
	Exenatide	CSK	135 nm	spherical	+6 mV	6%	55%	7%	-	-	-	50 µg/kg	min. 3 h °	[239]
	Liraglutide	CSK, Hemagglutinin, pHPMA	140 nm	-	+1.4 mV	-	80%	10%	-	-	-	540 µg/kg	min. 20 h *°	[240,241]
	Liraglutide	Eudragit^®^S100	253 nm	-	+25 mV	10%	72%	-	-	≈<10% in 2 h	≈60% in 48 h	100–400 µg/kg twice a day	-	[242]
Silica NPs	GLP-1	Aerosil^®^200, Eudragit^®^L100	308 nm	-	−26 mV	5%	-	36% p.o. vs. i.p.	77% p.o. vs. i.p.	≈35% in 2 h	≈80% in 1 h + ≈10% in 11 h	1 mg/kg	2 h °	[243]
	GLP-1	Chitosan	363 nm	irregular	+19 mV	17%	85%	-	-	≈20% in 2 h, ^→^	≈15% in 4 h	-	-	[206]
	GLP-1	Chitosan, polyarginine R9, HPMC acetate succinate, DPP4-inhibitor	320 nm before HPMC coating, afterw. ≈60 µm	spherical	+19 mV before HPMC coating	7.5% before HPMC coating	75% before HPMC coating	-	-	<5% in 2 h	≈40% in 30 min, no further release in 5.5 h	-	-	[225]
	GLP-1	Chitosan, HPMC acetate succinate, DPP4-inhibitor	400–500 nm	-	≈−30 mV	14% before HPMC coating	69% before HPMC coating	-	-	no	≈37% in 15 min, no further rel. in 1.75 h	200–250 µg/kg	min. 8 h *°	[180,244]
	GLP-1	Chitosan, HPMC acetate succinate, Human IgG Fc	603 nm	spherical	−2 mV	0.55%	30%	-	-	no, ^→^	≈40% in 15 min + ≈25% in 5.75 h	-	-	[245]
	Exenatide	Chitosan, THMP phosphonate	340–740 nm	-	pH 1.2 ≈ +20 mV, pH 6.8 = −11 mV	35% before chitosan coating	-	-	-	18% in 5 min, no further release in 24 h	≈25% in 5 min + ≈5% in 24 h	-	-	[246]
Lipid based NPs (SLNs)	GLP-1	Chitosan	224 nm	spherical	+13 mV	0.14%	57%	-	-	≈40% in 1 h, no release afterwards, ^→^	no	-	-	[206]
Lipid based NPs (NLCs)	Exenatide	Precirol^®^, Miglyol^®^, Tween^®^80, Poloxamer^®^188	161 nm	-	−20 mV	≈0.2%	87.5%	-	-	≈20% in 0.5 h, no further release in 1.5 h	≈100% in 0.5 h, no further release in 5.5 h	-	-	[247]
	Liraglutide	Precirol^®^, Miglyol^®^, Tween^®^80, Poloxamer^®^188	225 nm	-	−21 mV	≈0.2%	95.4%	-	-	≈20% in 0.5 h, no further release in 1.5 h	≈60% in 0.5 h, no further release in 5.5 h	-	-	[247]
Lipid based NPs (RM-LNCs)	Exenatide	Span^®^80, Labrafac^®^, Peceol^®^, Lipoid^®^, Solutol^®^, NaCl	224 nm	-	−3 mV	-	85%	4%	-	-	≈60% in 6 h	500 µg/kg	min. 2 h *°	[248]
	Exenatide	Span^®^80, Labrafac^®^, Peceol^®^, Lipoid^®^, Solutol^®^, NaCl, DSPE-PEG2000	213 nm	-	−9 mV	-	100%	-	-	-	≈75% in 6 h	500 µg/kg	min. 2 h *°	[249]
	Semaglutide	Span^®^80, Labrafac^®^, Peceol^®^, Lipoid^®^, Kolliphor^®^, NaCl	188 nm	-	−10 mV	-	90%	-	-	-	-	500 µg/kg	-	[250]
	Exenatide	Span^®^80, Labrafac^®^, Phospholipon^®^, Solutol^®^, NaCl, DSPE-PEG2000-FA	130 nm	spherical	−0.3 mV	-	79%	7%	-	≈20% in 2 h, ^→^	≈35% in 2 h + ≈5% in 4 h	100 µg/kg	min. 24 *°	[217]
Lipid based NPs (Liposomes)	Exendin-4	DOPC, DOTAP, chondroitin sulfate-g-glycocholic acid	229 nm	-	−31 mV	1.2%	74%	20%	-	-	51% in 24 h + 5% in 48 h	200 µg/kg	min. 72 h °	[251]
	Liraglutide	DSPC, Cholesterol, DOTAP, BSA, AT-1002	203 nm	spherical	2 mV	2%	85%	9% p.o. vs. i.j.	-	≈20% in 1 h, ≈5% in 1 h, ^→^	≈20% in 1 h + ≈10% in 7 h	540 µg/kg	min. 12 h *°	[252,253]
Lipid based NPs (SEDDS)	Exenatide	Sodium docusate, Cremophor^®^ EL, Labrafil^®^, Campul^®^PG 8, Propylene glycol	46 nm	-	−0.7 mV	-	-	15%	-	-	-	≈550 µg/kg	min. 4 h °	[254]
	Exenatide	*n*-octadecyl sulfate, Capmul^®^MCM EP, Captex^®^355, Kolliphor^®^RH40, Propylene glycol	30 nm	-	−17 mV	-	-	20%	-	-	-	300 µg/kg	min. 6 h °	[255]
Lipid based NPs (nanoemul.)	Exenatide	Tetraheptyl-ammoinium bromide, Capmul^®^MCM EP, Captex^®^355, Kolliphor^®^RH, Propylene glycol	26 nm	-	+10 mV	-	-	28%	-	-	-	300 µg/kg	min. 6 h °	[256]
	Exenatide	Span^®^80, Capric acid, Tween^®^80, Sodium citrate	251 nm	spherical	−51 mV	-	96.4%	24%	-	-	-	300 µg/kg	min. 12 h *	[257]
Lipid based NPs (RM-SEDDS)	Exenatide	Glycerol monolinoleate, Soybean phospholipid, Labrasol^®^, Cremophor^®^RH4, DOTAP, HA	309 nm	spherical	−1.7 mV	-	-	-	-	≈15% in 2 h, ^→^	≈20% in 1 h + ≈15% in 9 h	1000 µg/kg	min. 12 h *	[215]
Protamine NPs	Exenatide-zinc	Deoxycholic acid-LMWP, Glycocholic acid-poly (ethylene glycol)-b-polysialic acid copolymers, Enteric-coated capsules	140 nm	-	−22 mV	9%	78%	12%	-	-	-	300 µg/kg	min. 12 h °	[258]
Cyclodextrin NPs	Liraglutide	mPEG-DSPE 2000, dextran sulphate	101 nm	spherical	−35 mV	5%	100%	-	-	-	-	200 µg/kg	min. 2 h *	[259]
MOF NPs	Exendin-4	Iron chloride, 2-aminoterephthalic acid hexahydrate, Citric acid, Sodium bicarbonate, Enteric-coated capsules	≈160 nm	-	≈−7 mV	0.4%	87%	-	17%	-	≈70% in 4 h + ≈20% in 8 h	400 µg/kg	min. 10 h *°	[260]
Zein NPs	Exenatide	Phosphatidyl-choline, Cholic acid, Casein, Hypromellose phthalate	241 nm	spherical	−13 mV	-	79.7%	11%	18.6%	≈6% in 2 h, ^→^	≈30% in 2 h + ≈35% in 44 h	600 µg/kg	min. 24 h *°	[261]
	Liraglutide	Rhamnolipids R90, Cholic acid	160 nm	spherical	≈−17.5 mV	4.9%	76.8%	-	9.6%	≈18% in 2 h, ^→^	≈40% in 10 h + ≈30% in 36 h	4 mg/kg	min. 24 h *	[262]
	Liraglutide	Eudragit^®^RS100, Chitosan	239 nm	spherical	+41 mV	-	41.1%	71%	-	11.8% in 2 h, ^→^	3% in 4 h	200 µg/kg	min. 12 h °	[182]
Milk Exosomes	Semaglutide	DSPE-Hyd-PMPC, DSPC, DOTAP, Cholesterol, Enteric-coated capsules	145 nm	irregular	−2 mV	31%	32%	9%	11%	0% in 2 h, ^→^	≈8% in 6 h	300 µg/kg	min. 48 h °	[263]
Aminoclay NPs	Liraglutide	Citric acid, Eudragit^®^S100	370 nm	spherical	−2 mV	-	91%	-	-	≈30% in 2 h	≈70% in 1 h + ≈20% in 23 h	15 mg/kg	24 h °	[264]
	Semaglutide	Citric acid, Eudragit^®^S100	318 nm	spherical	−23.7 mV	-	93%	-	-	20% in 0.5 h, no further release in 1.5 h	62% in 0.5 h, no further release in 23.5 h	8 mg/kg	-	[265]
Bile Acid NPs	Liraglutide	Bile acid derivatives (TLC, LDC), PEG 200, Labrasol^®^	97 nm	spherical	+5 mV	-	98%	4%	-	34% in 2 h	-	10 mg/kg	min. 10 h °	[202]
	Liraglutide	Bile acid derivatives (DC, LDC), DDM, Enteric-coated capsules	76 nm	-	+5 mV	-	101%	5%	-	no	100% in 2 h	10 mg/kg	min. 24 h °	[266]

## Data Availability

No new data were created or analyzed in this study. Data sharing is not applicable to this article.

## Abbreviations

5-CNAC	8-(N-2-hydroxy-5-chlorobenzoyl)-amino-caprylic acid
ABHD12	αβ-hydrolase 12
Aib	aminoisobutyric acid
AMT(s)	advanced microbiome therapeutic(s)
ASBT(s)	apical sodium bile acid transporter(s)
AUC	area under the curve
BAT	brown adipose tissue
BMI	body mass index
bPEI	branched polyethyleneimine
BSA	bovine serum albumin
C/EBPs	CCAAT-enhancer-binding proteins
C10	sodium caprate
C8	sodium caprylate
CAGE	choline and geranic acid
CHONAC	choline salcaprozate
CKD	chronic kidney disease
COM NP(s)	complexed nanoparticle(s)
CPP(s)	cell penetrating peptide(s)
CV	cardiovascular
CYP	cytochrome P450
DAGL	diacylglycerol lipase
DC	deoxycholate
DDM	n-dodecyl-β-D-maltoside
DEX	dextran
DIS NP(s)	dispersed nanoparticle(s)
DLPC	dilauroyl phosphatidylcholine
DMG-PEG	1,2-dimyristoyl-rac-glycero-3-methoxy poly (ethylene glycol)-200
DOC	sodium docusate
DPP-4	dipeptidylpeptidase-4
DPPC	dipalmitoyl-sn-glycero-3-phosphocholine
DSPE	1,2-distearoyl-sn-glycero-3-phosphorylethanolamine
DSPE-Hyd-PMPC	1,2-distearoyl-sn-glycero-3-phosphorylethanolamine-hydrazone bond-poly(2-methacryloyloxyethyl phosphorylcholine)
EBMTs	endoscopic bariatric and metabolic therapies
ECD	extracellular domain
ECL	extracellular loop
EMA	European Medicines Agency
FA	folic acid
Fc region	fragment crystallizable region
FcRn	neonatal Fc receptor
FDA	Food and Drug Administration
GCA	glycocholic acid
*Gcg*	preproglucagon-gene
GCGR	glucagon receptor
GIP	glucose-dependent insulinotropic polypeptide
GIPR	glucose-dependent insulinotropic polypeptide receptor
GI-tract	gastrointestinal tract
GLP-1	glucagon-like peptide 1
GLP-1R	glucagon-like peptide 1 receptor
GLP-2	glucagon-like peptide 2
GPCRs	G-protein coupled receptors
GRAS	generally regarded as safe
GRK2	G protein-coupled receptor kinase 2
HA	hyaluronic acid
HPMC	hydroxypropyl methylcellulose
IgG	immunoglobulin G
IL(s)	ionic liquid(s)
INCL	intracellular loop
KLF(s)	kruppel-like transcription factor(s)
KO mice	knock-out mice
LAB	lactic acid bacteria
LDC	lysine conjugated deoxycholic acid
LMWP	low molecular weight protamine
LNC(s)	lipid nanocapsule(s)
lPEI	linear polyethylenimine
MASH	metabolic associated steatohepatitis
MC4R	melanocortin-4 receptor
MCFA(s)	medium-chain fatty acid(s)
MCT(s)	medium-chain triglyceride(s)
MOF	metal organic framework
MP(s)	microparticle(s)
NLC(s)	nanostructured lipid carrier(s)
NP(s)	nanoparticle(s)
NTCP(s)	sodium/taurocholate co-transporting polypeptide(s)
NTS	nucleus of the solitary tract
OGT	oral glucose test
PC	prohormone convertase
PCFT	proton-coupled folate transporter
PE(s)	permeation enhancer(s)
PEG	polyethylene glycol
PGC-1α	peroxisome proliferator-activated receptor-gamma coactivator 1 alpha
pHPMA	poly-N-(2-hydroxypropyl)-methacrylamide
PLA	polylactic acid
PLGA	poly(D,L-lactide-co-glycolide)
PPAR-γ	peroxisome proliferator activated receptor gamma
PRDM16	PR domain containing 16
PSi nanoparticles	porous silicon nanoparticles
PYY	peptide YY
RM(s)	reverse micelle(s)
RYGB	Roux-Y gastric bypass
SEDDS(s)	self-emulsifying drug delivery system(s)
SG	sleeve gastrectomy
SIP	structure inducing probe
SLN(s)	solid lipid nanoparticle(s)
SNAC	sodium N-[8-(2-hydroxybenzoyl)amino]caprylate
SOS	sodium n-octadecyl sulfate
T2DM	type 2 diabetes mellitus
Tf	transferrin
TfR	transferrin receptor
THA	tetraheptylammonium bromide
TLC	taurolithocholate
TMC	trimethyl chitosan
TMD	transmembrane domain
TNFα	tumor necrosis factor α
WAT	white adipose tissue
WHO	World health organization
ZDF	Zucker diabetic fatty
γGlu-2xOEG	γ-Glutamyl-bis(oligoethylene glycol), γ-Glutamyl-bis(oligoethylene glycol)
γ-PGA	poly(γ-glutamic acid)
